# Bone Marrow-Resident Vδ1 T Cells Co-express TIGIT With PD-1, TIM-3 or CD39 in AML and Myeloma

**DOI:** 10.3389/fmed.2021.763773

**Published:** 2021-11-08

**Authors:** Franziska Brauneck, Pauline Weimer, Julian Schulze zur Wiesch, Katja Weisel, Lisa Leypoldt, Gabi Vohwinkel, Britta Fritzsche, Carsten Bokemeyer, Jasmin Wellbrock, Walter Fiedler

**Affiliations:** ^1^Department of Oncology, Hematology and Bone Marrow Transplantation With Section Pneumology, Hubertus Wald University Cancer Center, University Medical Center Hamburg-Eppendorf, Hamburg, Germany; ^2^Infectious Diseases Unit, I. Department of Medicine, University Medical Center Hamburg-Eppendorf, Hamburg, Germany; ^3^University Cancer Center Hamburg (UCCH)-Biobank, Department of Oncology, Hematology and Bone Marrow Transplantation With Section Pneumology, Hubertus Wald University Cancer Center, University Medical Center Hamburg-Eppendorf, Hamburg, Germany

**Keywords:** (Vδ1) γδ T cells, TIGIT, PD-1, CD39, AML, myeloma

## Abstract

**Background:** γδ T cells represent a unique T cell subpopulation due to their ability to recognize cancer cells in a T cell receptor- (TCR) dependent manner, but also in a non-major histocompatibility complex- (MHC) restricted way via natural killer receptors (NKRs). Endowed with these features, they represent attractive effectors for immuno-therapeutic strategies with a better safety profile and a more favorable anti-tumor efficacy in comparison to conventional αβ T cells. Also, remarkable progress has been achieved re-activating exhausted T lymphocytes with inhibitors of co-regulatory receptors e.g., programmed cell death protein 1 (PD-1), T cell immunoreceptor with Ig and ITIM domains (TIGIT) and of the adenosine pathway (CD39, CD73). Regarding γδ T cells, little evidence is available. This study aimed to immunophenotypically characterize γδ T cells from patients with diagnosed acute myeloid leukemia (AML) in comparison to patients with multiple myeloma (MM) and healthy donors (HD).

**Methods:** The frequency, differentiation, activation, and exhaustion status of bone marrow- (BM) derived γδ T cells from patients with AML (*n* = 10) and MM (*n* = 11) were assessed in comparison to corresponding CD4^+^ and CD8^+^ T cells and peripheral blood- (PB) derived γδ T cells from HDs (*n* = 16) using multiparameter flow cytometry.

**Results:** BM-infiltrating Vδ1 T cells showed an increased terminally differentiated cell population (TEMRAs) in AML and MM in comparison to HDs with an aberrant subpopulation of CD27^−^CD45RA^++^ cells. TIGIT, PD-1, TIM-3, and CD39 were more frequently expressed by γδ T cells in comparison to the corresponding CD4^+^ T cell population, with expression levels that were similar to that on CD8^+^ effector cells in both hematologic malignancies. In comparison to Vδ2 T cells, the increased frequency of PD-1^+^-, TIGIT^+^-, TIM-3^+^, and CD39^+^ cells was specifically observed on Vδ1 T cells and related to the TEMRA Vδ1 population with a significant co-expression of PD-1 and TIM-3 together with TIGIT.

**Conclusion:** Our results revealed that BM-resident γδ T cells in AML and MM express TIGIT, PD-1, TIM-3 and CD39. As effector population for autologous and allogeneic strategies, inhibition of co-inhibitory receptors on especially Vδ1 γδ T cells may lead to re-invigoration that could further increase their cytotoxic potential.

## Introduction

Although γδ T cells represent a relatively small subset within all T lymphocytes (1–5%) (1, 2), they have a unique property to recognize cancer cells in a T cell receptor- (TCR) dependent manner but also in a non- major histocompatibility complex- (MHC) restricted way via their expression of natural killer cell receptors (NKRs) (3). In immuno-oncology, γδ T cells represent a novel attractive effector population for immuno-therapeutic strategies such as chimeric antigen receptor T cells (CAR-T cells) or bispecific T cell engagers (BiTEs).

In humans, γδ T cells can be differentiated into two major subsets by their expression of the Vδ chain. Vδ2 cells constitute the major circulating γδ T cell population in the peripheral blood (PB) whereas the Vδ1 subpopulation is enriched in the peripheral tissue (4, 5). Both γδ subpopulations exhibit cytotoxic capacities mediated by TCR- and natural killer group 2D (NKG2D) receptor signaling via production of the effector cytokines interferon (IFN)-γ, tumor necrosis factor (TNF)-α and soluble mediators such as perforin or granzymes (5–7). Moreover, γδ T cells can induce dendritic cell (DC) maturation via secretion of TNF-α (8, 9). They also have an antigen-presenting capacity by MHC-II loading and expression (10). Recent data also demonstrate a phagocytic potential of γδ T cells through expression of the scavenger receptor CD36 which is dependent on the transcription factor CCAAT-enhancer-binding protein α (C/EBPα) (11).

In oncology, both the Vδ1 and Vδ2 T cells have been described to exert pleiotropic effector functions: as mentioned above the tumor-infiltrating IFNγ-producing γδ T cell fraction has shown cytotoxic effects in solid and hematological malignancies (7, 12, 13), whereas interleukin (IL)-17- and galectin 1-secreting γδ T cells promote tumor growth and the recruitment of immunosuppressive myeloid cells (14, 15). By analyzing molecular profiles of expression signatures of 18.000 tumors from 39 different malignancies, including AML and MM with overall survival data, Gentles et al. identified tumor-infiltrating γδ T cells as the leukocyte subset with the most significant favorable cancer-wide prognostic relevance (16).

First clinical observations of patients with refractory/relapsed AML transplanted with haploidentical γδ T cells showed that γδ T cells can induce clinical anti-tumor effects (17, 18). Furthermore, preclinical data provide support for this contention: AML cells were efficiently killed by Vδ2 T cells *in vitro* (3, 6, 19). In an AML xenograft model, Vδ2 T cells traffic to the BM and have been shown to slow the progression of the disease (6). Also, in MM it has been recently published that impairment of Vδ2 T cell functions (including decreased proliferation and cytotoxicity) was already detectable in monoclonal gammopathy of undetermined significance (MGUS) (20).

Compared to Vδ2 T cells, Vδ1 T cells account for a small proportion of γδ T cells (1). Although most of the studies investigating the antitumoral effects of γδ T cells focus on Vδ2 T cells, it is becoming increasingly evident that Vδ1 T cells play a critical role in the anti-tumor functionality (21, 22). Enhanced reconstitution of γδ T cells following allogeneic hematopoietic stem cell transplantation (aHSCT) was associated with improved survival in patients with AML (23). Cordova et al. demonstrated that Vδ1 tumor-infiltrating lymphocyte- (TIL-) derived cells outperformed Vδ2 TILs in *in vitro* tumor cytotoxicity assays in malignant melanoma (24). In AML, increased levels of cytomegalovirus- (CMV) specific Vδ1 cells were associated with a reduced relapse probability. *In vitro*, these Vδ1 cells demonstrated increased cytotoxicity against AML cells, which could be further enhanced following CMV reactivation (19, 25). Additionally, Knight et al. showed a significant cytotoxicity of Vδ1 T cells for MM by lysis against patients' CD38^+^CD138^+^ BM-derived plasma cells *in vitro* (26).

First phenotypic analyses of BM-derived Vδ2 T cells from AML patients showed an increased subpopulation with a memory phenotype that was associated with a reduced capacity for expansion and cytotoxicity (6).

This study is focused on programmed cell death protein-1 (PD-1), the novel receptor T cell Ig and ITIM domain (TIGIT), the T cell immunoglobulin and mucin domain-containing protein 3 (TIM-3) and the ectonucleotidases ectonucleoside triphosphate diphosphohydrolase-1 (CD39) and ecto-5'-nucleotidase (CD73). All of these checkpoints are involved in αβ T lymphocyte dysfunction in cancer (27–29). TIGIT, PD-1 and TIM-3 are co-inhibitory receptors highly expressed by exhausted T cells in chronic infections and cancer (27, 30, 31). Inhibition of these receptors have shown increased proliferation and cytotoxic efficiency *in vitro* (27, 30–32). Recently, inhibition of the adenosine-generating enzymes CD39 and CD73 showed anti-tumor immunity through multiple mechanisms, including enhancement of T cell and natural killer- (NK) cell function (33, 34). It is well-acknowledged that multiple co-expression of inhibitory receptors and suppression of inflammatory cues by CD39 enzymatic overactivity are highly associated with the severity of T cell dysfunction (35, 36), thus representing an important feature of T cell exhaustion (37). Regarding the (co)-expression of these checkpoints on γδ T cells, little is known. Jin et al. showed for the first time that TIGIT is also expressed by regulatory Forkhead-Box-Protein P3 (FOXP3)^+^ γδ T cells in AML (38). Moreover, it has been presented that tumor-infiltrating γδ T cells express the ectonucleotidases CD39 and CD73 and may be dysfunctional via the activation of adenosine-mediated pathways (39, 40).

Since circulating γδ T cells in the peripheral blood have been more elucidated, the present study provides an extensive immunophenotypic characterization of γδ T cells derived from the bone marrow of patients with untreated newly diagnosed acute myeloid leukemia or multiple myeloma. We focus on the comparison of the Vδ1- and Vδ2-cell phenotypes, including differentiation and expression of immune checkpoints and metabolic molecules that may dampen antitumor immunity in patients with acute myeloid leukemia in comparison to patients with myeloma and healthy volunteers.

## Materials and Methods

### Patient Cohorts

Bone marrow-derived aspirates were collected from patients with newly diagnosed non-M3 AML (*n* = 10) and patients with multiple myeloma (*n* = 11) before the start of intensive chemotherapy treatment, and peripheral blood specimens from age-matched healthy donors (HD, *n* = 16) after written informed consent in accordance with the Declaration of Helsinki and approval by the local ethics board of the Ärztekammer Hamburg (PV3469 and PV5119). The median age of the AML patient cohort was 70 years (range 43–86), the median age of the MM patient cohort 61.5 years (range 55–86), and the median age of the healthy donors was 60 years (range 27–71) (Supplementary Table 1).

### Multiparameter Flow Cytometry

For multiparametric flow cytometry analysis (MFC), cryopreserved BM mononuclear cells from patients with CD117^+^CD33^+^ AML, from patients with CD38^+^CD138^+^ MM and peripheral blood (PB) mononuclear cells from HDs were thawed and counted. After washing with PBS and FCR blocking (FcR blocking reagent, human, Miltenyi Biotec), mononuclear cells were stained with the LIVE/DEAD™ Fixable Near-IR dye (Thermo Fisher) according to the manufacturer's protocol for exclusion of dead cells. Afterwards, cells were washed and incubated for surface staining with appropriate fluorochrome-conjugated antibodies, including anti-CD3 (OKT3), anti-CD4 (RPA-T4), anti-CD8 (RPA-T8), anti-CD33 (P67.6), anti-CD117 (104D2), anti-CD38 (HIT2), anti-CD138 (MI15), anti-γδ TCR (B1), anti-Vδ1 TCR (REA173), anti-Vδ2 TCR (REA771), anti-CD45RA (HI100), anti-CD27 (O323), anti-CD19 (HIB19), anti-CD56 (HCD56), anti-PD-1 (EH12.2H7), anti-TIGIT (A15153G), anti-TIM-3 (F38-2E2), anti-CD39 (A1), anti-CD73 (AD2), and anti-HLA-DR (L243) for 20 min at room temperature in the dark. Subsequently, samples were fixed with 0.5% paraformaldehyde (Sigma Aldrich) and incubated for 15 min at 4°C in the dark. Antibodies were obtained from Biolegend, BD Biosciences or Miltenyi Biotec. Compensation controls were measured using single-stained Comp Beads (Anti-Mouse Ig,κ/Negative Control Compensation Particles Set, BD Biosciences). For live/dead compensation, Comp Beads stained with anti-CD19 (APC Cy-7, BioLegend) were applied. All samples were run on a BD FACSymphony A3 with FACS Diva version 8 (BD Biosciences).

### T-Distributed Stochastic Neighbor Embedding Analyses

Individual donor FCS files were imported into FlowJo version 10.5.2. A subset of 3,000 cells were selected for each donor at random and merged into a single expression matrix prior to tSNE analysis. The following channels were removed from the expression matrix to only include protein markers in tSNE analysis: viability, CD19, CD56, AML lineage markers (CD33, CD117), MM lineage markers (CD38, CD138), offset, residual, and time. A total of 12,000 cells and 14 markers were used to create a tSNE map of the PB- and BM- derived γδ T cells from HDs and patients with AML and MM. A perplexity parameter of 30 and iteration number of 550 was used for applying the dimensionality reduction algorithm. The output was in the form of a matrix with two columns corresponding to tSNE dimension 1 and dimension 2. tSNE maps were generated by plotting each event by its tSNE dimensions in a dot-plot. Intensities for markers of interest were overlaid on the dot-plot to show the expression of those markers on different cell islands.

### Statistical Analysis

All flow cytometric data were analyzed using FlowJo version 10.5.2. software (Treestar). Statistical analysis was carried out using Prism 7.0 software (GraphPad Software). Groups were tested for normal distribution with the Kolmogorov-Smirnov test. Non-normally distributed data were analyzed by the Mann-Whitney test for two unpaired groups, the Wilcoxon test for two paired groups, respectively. Pearson's correlation and Spearman's rank correlation coefficient were applied for bivariate correlation analysis. Frequencies in the text are described as medians unless stated otherwise (as indicated in the figure legend). *P*-values below 0.05 were considered significant, where ^*^, ^**^, and ^***^ indicate *p*-values between 0.01–0.05, 0.001–0.01 and 0.0001–0.001, respectively.

## Results

### BM-Derived Vδ1 T Cells of AML and MM Patients Show a Shift Toward Effector Memory- and Terminally Differentiated Memory Cells

**Figure 1:**
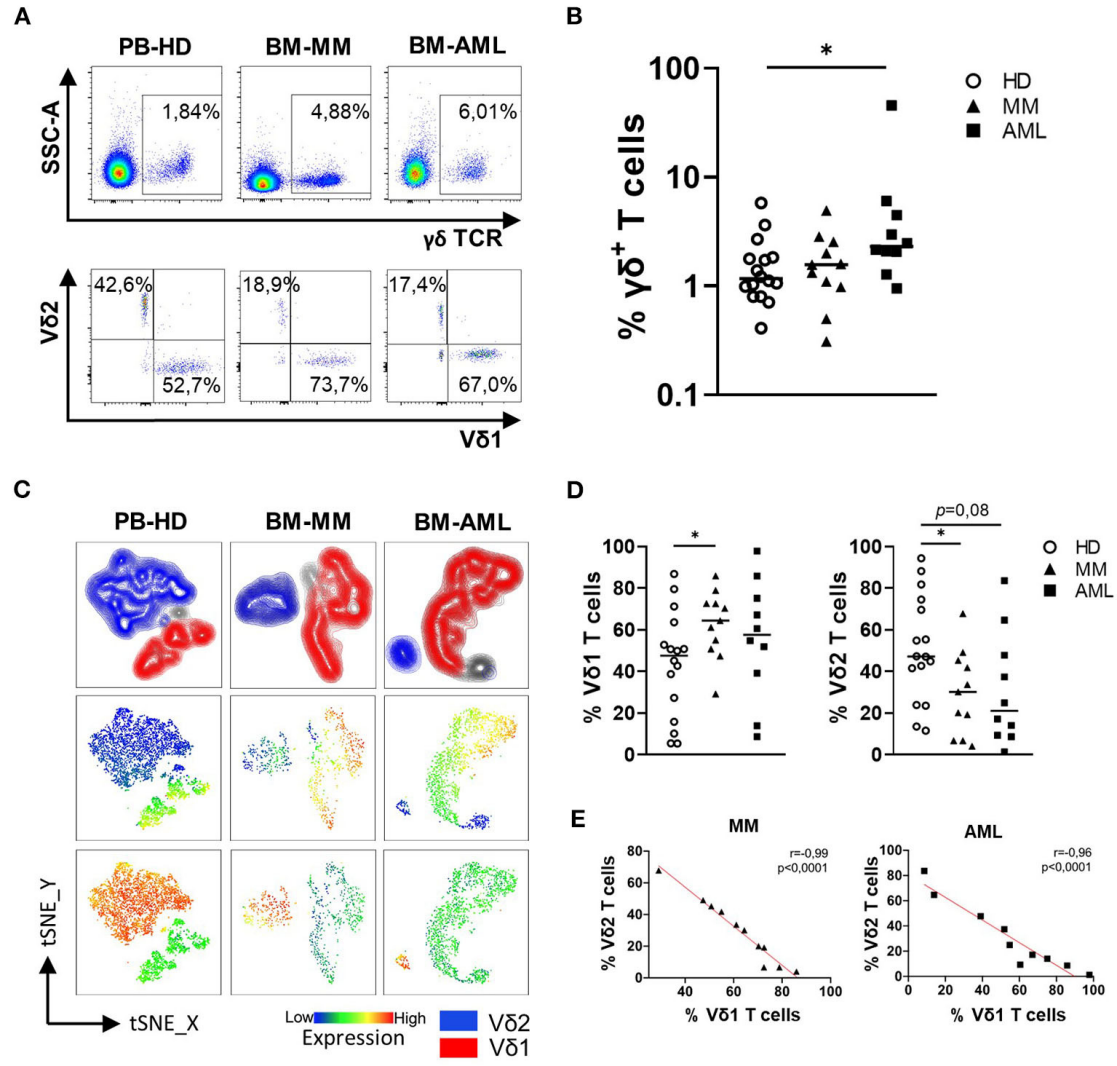
The increased frequency of Vδ1 γδ T cells is associated with reduced infiltration of Vδ2 γδ T cells in AML and MM. Flow cytometric analysis of the co-expression of the γδ TCR and the Vδ1 and Vδ2 receptor on CD3^+^ T cells was performed for bone marrow (BM) samples from patients with AML (AML, black rectangles, *n* = 10), MM patients (MM, black triangles, *n* = 11), and peripheral blood (PB)-derived mononuclear cells from healthy donors (HD, white circles, *n* = 16). **(A)** Representative flow cytometry data show γδ T cells, Vδ1, and Vδ2 γδ T cells. **(B)** Summary data illustrate the frequency of γδ T cells in HD, AML, and MM. *P*-values were obtained by the Mann-Whitney test. *P < 0.05, **P < 0.01, ***P < 0.001. **(C)** t-distributed stochastic neighbor embedding (tSNE) analysis delineate the distribution of Vδ1 and Vδ2 γδ T cells within the total γδ T cells in BM aspirates from four HDs (left graphs), four patients with MM (middle graphs), and four patients with AML (right graphs). **(D)** Summary data show the frequency of Vδ1 and Vδ2 T cell subpopulations. *P*-values were obtained by the Mann-Whitney test. *P < 0.05, **P < 0.01, ***P < 0.001. **(E)** Correlative analysis of the expression of the Vδ1 and Vδ2 receptors was performed for BM-derived aspirates of the MM and AML patients. Pearson's test was used to test for correlations.

γδ T cells were analyzed in the BM from patients with AML (*n* = 10) and MM (*n* = 11) in comparison to the PB from age-matched HDs (*n* = 16). For gating strategy see Supplementary Figure 1. The study included aspirates of patients with typical phenotypes namely CD33^+^CD117^+^ for AML and CD38^+^CD138^+^ for MM aspirates. This selection of patients enabled us to differentiate tumor cells from further immune cell populations in the BM. Our studies revealed a significantly increased frequency of γδ T cells in AML in comparison to HD. In contrast, this was not observed for MM (AML vs. HD *p* = 0.014; MM vs. HD *p* = 0.707, MM vs. AML *p* = 0.085; Figures 1A,B), implying differences in the immune response between both entities. Additional characterization of the γδ T cells was performed to investigate the immunophenotype of these cells in more detail. As illustrated in the t-distributed stochastic neighbor embedding (tSNE) analysis (Figure 1C), the distribution of Vδ1 and Vδ2 γδ T cells differed between HD, AML and MM: Vδ2 T cells were more prevalent in the PB in HDs compared to the BM compartment in AML and MM (Figure 1D). The fraction of BM-infiltrating Vδ1 T cells was significantly increased in MM patients relative to HDs (*p* = 0.035; Figure 1D), but not in patients with AML (*p* = 0.220; Figure 1D). Due to a shift of γδ subpopulations in the BM in AML and MM patients compared to HDs, decreased levels of Vδ2 T cells were correlated with increased frequencies of Vδ1 T cells in BM of both malignancies (AML *r* = −0.96, *p* < 0.0001 and MM *r* = −0.98, *p* < 0.001; Figure 1E), indicating homing of Vδ1 T cells into the site of tumor development. To validate that these observations are not site-associated but malignancy-associated, we compared mononuclear cells derived from paired PB and BM aspirates of 9 AML patients. Our analyses revealed no differences regarding the total fraction of γδ T cells and the distribution of Vδ1 and Vδ2 γδ T cells between paired PB and BM (Supplementary Figure 2A).

Next, γδ T cells were subdivided into the following subpopulations regarding their differentiation status based on the expression of CD27 and CD45RA: Naïve (NA = CD27^+^CD45RA^+^), Central Memory (CM = CD27^+^CD45RA^−^), Effector Memory (EM = CD27^−^CD45RA^−^), Terminally Differentiated Memory Cells (TEMRA = CD27^−^CD45RA^+^), and a subset of TEMRA cells characterized by CD27^−^CD45RA^++^ expression (for gating strategy see Supplementary Figure 1).

**Figure 2:**
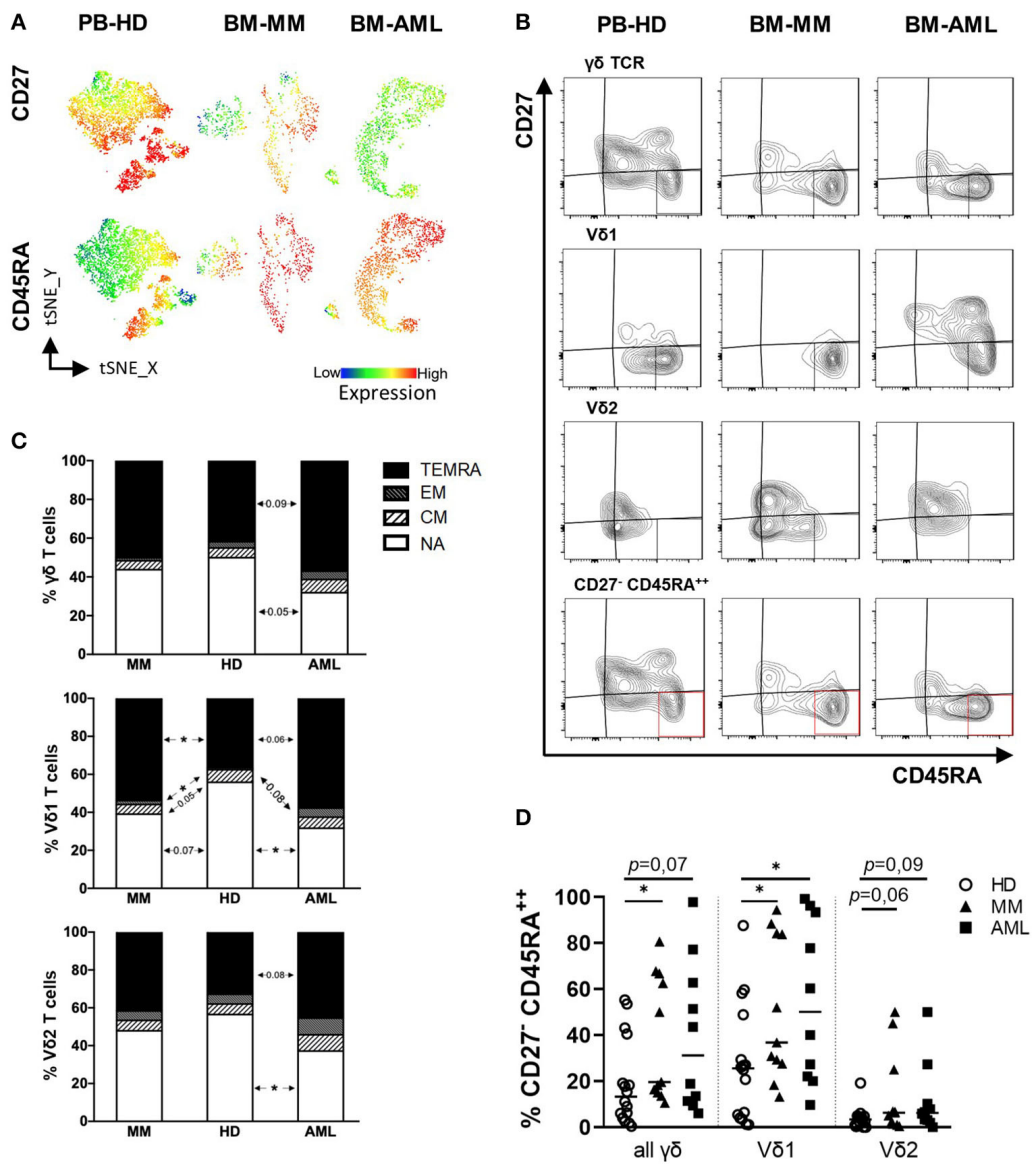
Vδ1 γδ T cells from patients with AML and MM show a shift toward increased EM and TEMRA differentiation. γδ T cell differentiation was analyzed by expression of CD45RA and CD27 for healthy donors (HD, white circles, *n* = 16), patients with multiple myeloma (MM, black triangles, *n* = 11) and newly diagnosed AML patients (AML, black rectangles, *n* = 10). CD27^+^CD45RA^+^: naïve (NA); CD27^+^CD45RA^−^: central memory (CM); CD27^−^CD45RA^+^: effector memory (EM); CD27^−^CD45RA^+^: terminally differentiated (TEMRA). **(A)** T-distributed stochastic neighbor embedding (tSNE) analysis illustrate the differentiation status of Vδ1 and Vδ2 γδ T cells within the total γδ T cells in BM aspirates from four HDs (left graphs), four patients with MM (middle graphs), and four patients with AML (right graphs). **(B)** Representative flow cytometry data show the differentiation of γδ T cells. **(C)** Summary data are demonstrating the distribution of the differentiation status of γδ T cells. The upmost graph shows the subsets of all γδ T cells, followed by Vδ1 and Vδ2 cells only. **(D)** Displays the frequency of CD27^−^ CD45RA^++^ cells in all γδ T cells and Vδ1 and Vδ2 only. *P*-Values were obtained by the Mann-Whitney test. *P < 0.05, **P < 0.01, ***P < 0.001.

As in the previous analyses Vδ1 cells have been identified as the predominant γδ population in the BM of AML and MM patients, further tSNE analyses showed a prevailing expression of CD45RA in AML and MM mainly in the Vδ1 T cell population (Figure 2A). Further summary analyses revealed an increased frequency of Vδ1 T cells within the EM- and TEMRA compartment in AML and MM in comparison to HDs (HD vs. AML *p* = 0.084 and *p* = 0.061; HD vs. MM *p* = 0.026 and *p* = 0.049; Figures 2B,C). This shift of Vδ1 T cells was not observed for the total γδ T cell population (Figures 2B,C, upper panel) and also not within the Vδ2 subpopulation (Figures 2B,C, lower panel). Moreover, the CD27^−^CD45RA^++^ population which has been described as a dysfunctional subpopulation with limited proliferation capacity (41) was significantly increased within the BM-derived Vδ1 T cells in AML and MM in comparison to HDs (HD vs. AML *p* = 0.036; HD vs. MM *p* = 0.026, respectively, Figure 2D).

Taken together, our data show an increased frequency of EM, TEMRA, and CD27^−^CD45RA^++^ Vδ1 T cells in the BM from patients with AML or MM diagnosis providing the rationale to further analyze this cell population.

### BM-Derived γδ T Cells From Patients With AML and MM Express PD-1, TIGIT, TIM-3, and CD39

**Figure 3:**
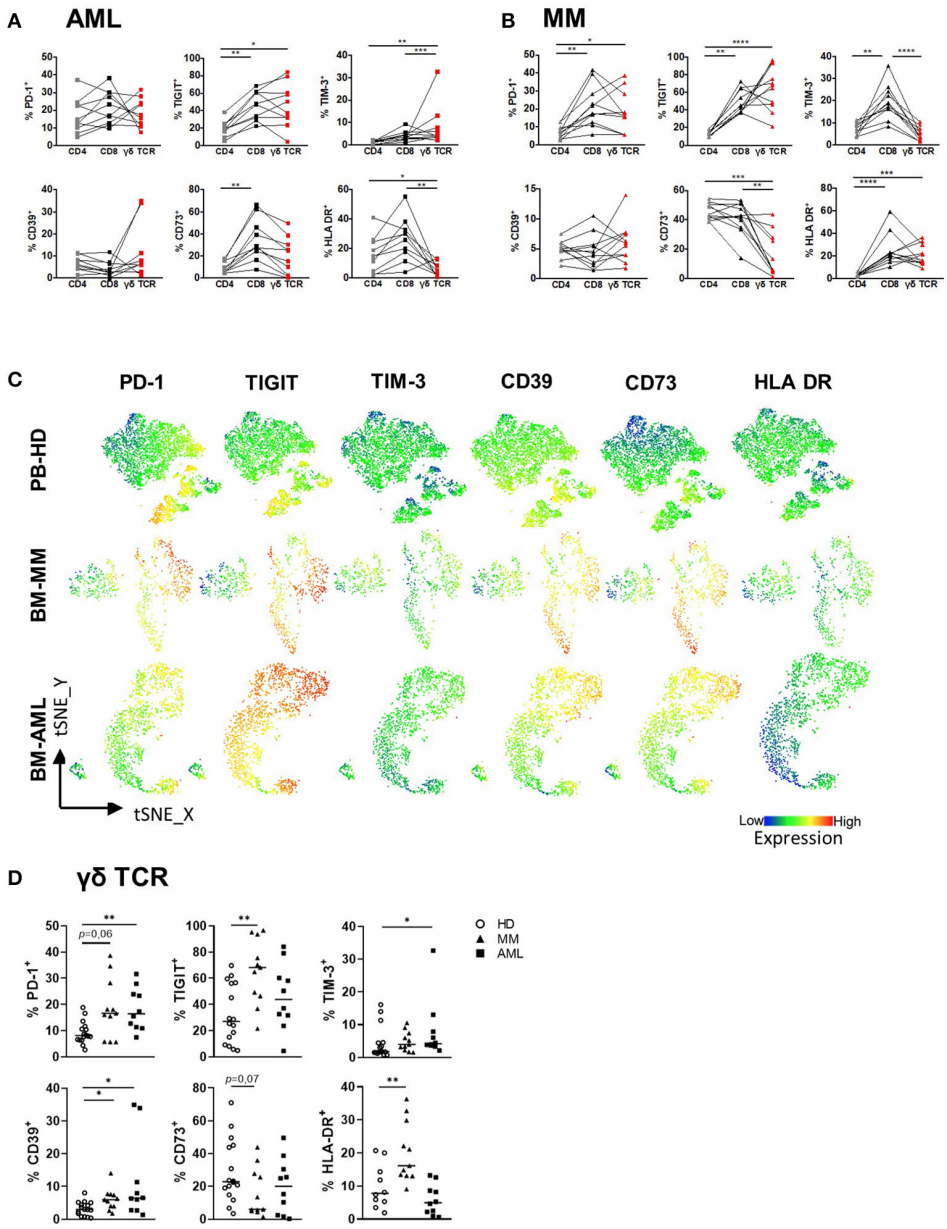
PD-1, TIGIT, TIM-3, and CD39 are expressed by γδ T cells in AML and MM. The surface expression of PD-1, TIGIT, TIM-3, CD39, CD73, and HLA-DR was investigated on CD4^+^, CD8^+^, and γδ T cells by using multicolor flow analysis for healthy donors (HD, *n* = 16), patients with multiple myeloma (MM, *n* = 11), and newly diagnosed AML patients (AML, *n* = 10). **(A,B)** Summary data illustrating the frequency of PD-1, TIGIT, TIM-3, CD39, CD73, and HLA-DR on CD4^+^ cells (gray squares/triangles), CD8^+^ T cells (black squares/triangles), and γδ T cells (red squares/triangles). *P*-values were obtained by the ANOVA and Friedmann test. *P < 0.05, **P < 0.01, ***P < 0.001, ****P < 0.0001. **(C)** T-distributed stochastic neighbor embedding (tSNE) analysis demonstrate the distribution of PD-1, TIGIT, TIM-3, CD39, CD73, and HLA-DR on Vδ1 and Vδ2 γδ T cells within the total γδ T cells in BM aspirates from four HDs (upper graphs), four patients with MM (middle graphs), and four patients with AML (lower graphs). **(D)** Summary data show the expression of PD-1, TIGIT, TIM-3, HLA-DR, CD39, and CD73 on γδ T cells in HD (HD, white circle) vs. MM (black triangles) vs. AML (black rectangles). *P*-values were obtained by the Mann-Whitney test. *P < 0.05, **P < 0.01, ***P < 0.001.

Several studies have identified the expression of co-regulatory receptors and ectonucleotidases as characteristic features of altered αβ T cell function (42), but little is known about their surface expression and functional relevance on γδ T cells. For comprehensive immunophenotyping of γδ T cells, we compared the expression of the co-inhibitory molecules PD-1, TIGIT, TIM-3, the ectonucleotidases CD39 and CD73 and the human leukocyte antigen DR isotype (HLA-DR, which is expressed by activated T cells) between BM-derived γδ T cells from patients with AML and MM with that from corresponding CD4^+^ and CD8^+^ αβ T cells from the same patients. For AML, our analyses revealed an increased expression of TIGIT and TIM-3 on γδ T cells and CD8^+^ T cells in comparison to CD4^+^ T cells (γδ T cells vs. CD4: for TIGIT *p* = 0.01, for TIM-3 *p* < 0.001, and CD8 vs. CD4: for TIGIT *p* = 0.006, for TIM-3 *p* = 0.01; Figure 3A). In MM, a higher frequency of PD-1, TIGIT, and HLA-DR was observed within the γδ T cell and also the CD8^+^ T cell population in comparison to the corresponding CD4^+^ T cells (γδ T cells vs. CD4: for PD-1 *p* = 0.029, for TIGIT *p* < 0.0001 and for HLA-DR *p* = 0.0008; and CD8 vs. CD4: for PD-1 *p* = 0.0022, for TIGIT *p* = 0.002 and for HLA-DR *p* < 0.0001; Figure 3B). In MM, the frequency of CD73^+^ cells was significantly lower in the γδ T cell population in comparison to the CD4^+^ and CD8^+^ population (*p* = 0.0005 and *p* = 0.007 respectively; Figure 3B). Next, we compared the expression of the co-regulatory receptors in AML and MM with that from age-matched PB of HDs (for gating strategy see Supplementary Figure 3). Performing tSNE analyses, our data revealed intense regions of PD-1^+^, TIGIT^+^, TIM-3^+^, and CD39^+^ γδ T cells that differed in AML and MM from that in HDs (Figure 3C). Also, our comprehensive analyses showed an increased frequency of γδ T cells expressing PD-1 and CD39 in both neoplasias in comparison to HDs (Figure 3D). Furthermore, in AML TIM-3^+^ γδ T cells were more frequent in comparison to HDs (*p* = 0.035, Figure 3C) whereas in MM, γδ T cells showed increased levels of TIGIT^+^ –and HLA-DR^+^ cells (*p* = 0.002 and *p* = 0.006, respectively; Figure 3D). Analyzing the median fluorescence intensity (MFI), the same results were detected (Supplementary Figure 4A).

Overall, γδ T cells exhibit higher frequencies of co-regulatory receptors in comparison with CD4^+^ T cells, but similar to that expressed by CD8^+^ T cells. In contrast to HDs, γδ T cells in the BM from patients with AML and MM showed an increased expression of the co-inhibitory molecules PD-1, TIGIT, TIM-3 or CD39.

### Vδ1 T Cells Exhibit the Highest Frequency of Co-inhibitory Receptors in AML and MM

In our phenotypic comparisons of BM-derived γδ T cells, PD-1, TIGIT, TIM-3, and CD39 emerged as receptors expressed by γδ T cells in AML and MM. We thus analyzed receptor expression on different γδ T cell subpopulations.

**Figure 4:**
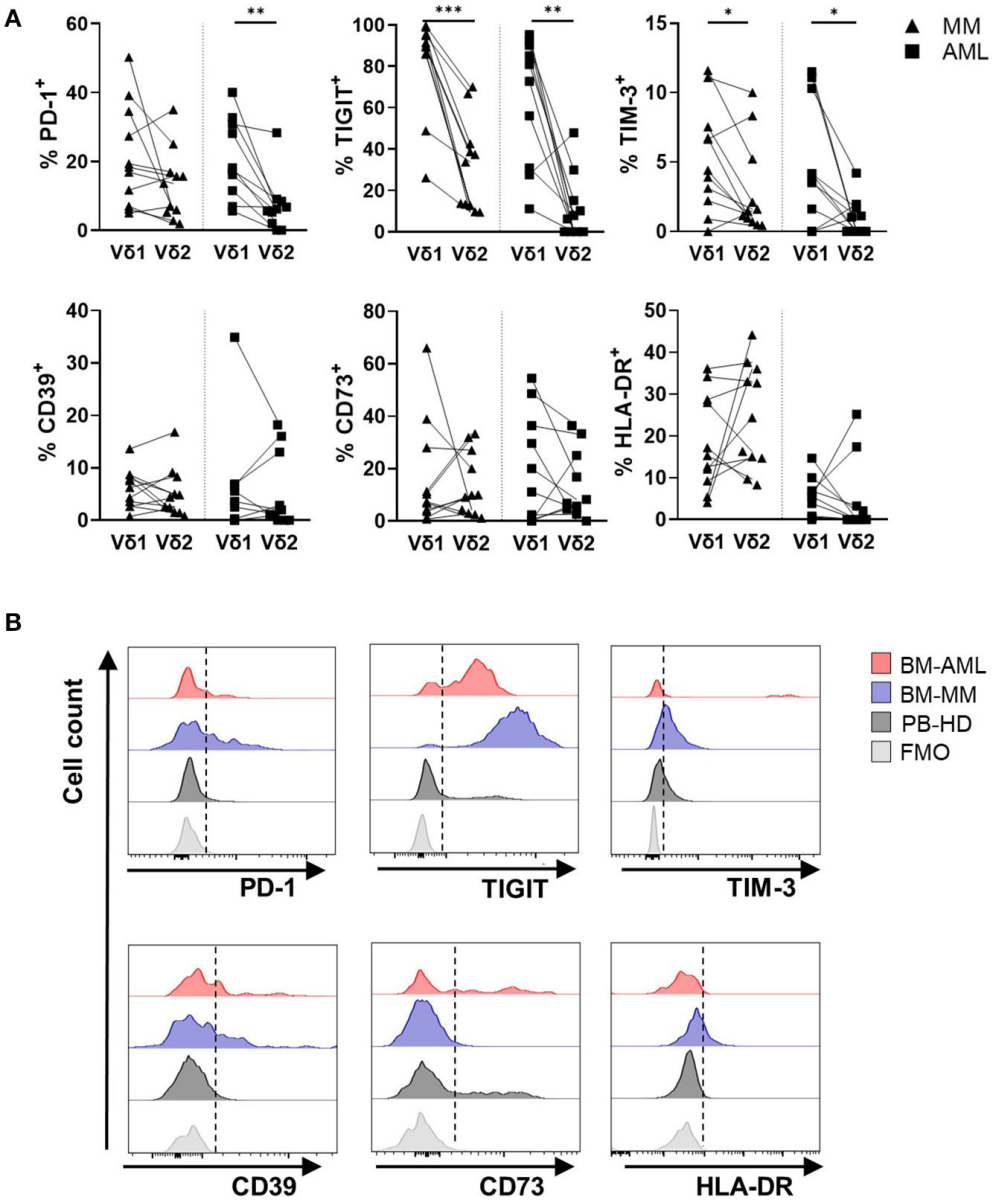
Vδ1 γδ T cells highly express PD-1, TIGIT, TIM-3, but not CD39, CD73, and HLA-DR in AML and MM. The surface expression of PD-1, TIGIT, TIM-3, CD39, CD73, and HLA-DR was compared between Vδ1 and Vδ2 T cells in bone marrow (BM) aspirates from patients with multiple myeloma (MM, black triangles, *n* = 11) and newly diagnosed AML patients (AML, black rectangles, *n* = 10). **(A)** Summary data show the paired distribution of PD-1, TIGIT, TIM-3, CD39, CD73, and HLA-DR expression in Vδ1 and Vδ2 T cells per patient. *P*-values were obtained by the Wilcoxon matched-pairs signed-rank test. *P < 0.05, **P < 0.01, ***P < 0.001. **(B)** Representative flow cytometry histograms demonstrate the expression of PD-1, TIGIT, TIM-3, CD39, CD73, and HLA-DR on γδ T cells in healthy donors (HD, dark gray), multiple myeloma patients (MM, blue), and newly diagnosed AML patients (AML, red) in comparison to the fluorescence-minus-one control (FMO, gray).

In both malignancies, TIGIT^+^ and TIM-3^+^ cells were significantly more frequent among the Vδ1 subpopulation in comparison to the Vδ2 subpopulation (AML: *p* = 0.006, *p* = 0.014 and MM: *p* = 0.001, *p* = 0.042; Figures 4A,B). Additionally, the PD-1 expression was increased on the Vδ1 cells in AML (*p* = 0.002, Figures 4A,B). Apart from TIGIT, the MFIs of TIM-3- and PD-1 MFIs were also significantly increased on the Vδ1–in comparison to the Vδ2 cells (Supplementary Figures 4B–D). Again, we compared mononuclear cells derived from paired PB and BM aspirates of 9 AML patients, to exclude that our data are compartment- and not tumor-associated. The frequencies of PD-1^+^, TIGIT^+^, TIM-3^+^, CD39^+^, and CD73^+^ (Vδ1) γδ T cells were similar in both compartments (Supplementary Figure 2B).

Regarding the differentiation status, CM γδ T cells showed higher frequencies of PD-1^+^ cells, whereas EM and TEMRA γδ T cells exhibit increased amounts of TIGIT and TIM-3 (Supplementary Figure 5). As the Vδ1 population in AML and MM showed a significant shift toward TEMRA and CD27^−^CD45RA^++^ differentiation, we further compared the receptor expression on these differentiation subgroups between paired Vδ1 and Vδ2 cells. In AML and MM, the frequency of TIGIT^+^ and TIM-3^+^ cells was higher on the TEMRA Vδ1 than on the TEMRA Vδ2 T cell subset (Supplementary Figure 6). Within the TEMRA Vδ1 T cell population, the aberrant population of CD27^−^CD45RA^++^ T cells showed the highest frequency of PD-1^+^-, TIGIT^+^-, TIM-3^+^-, and CD39^+^ cells in AML (Supplementary Figure 7). In MM, this was only the case for TIM-3 expression (Supplementary Figure 7), whereas CD73 was completely downregulated in these cells (Supplementary Figure 7).

Taken together, in both malignancies in contrast to HDs, the co-inhibitory receptors PD-1, TIGIT, and TIM-3 were expressed by the Vδ1 T cell subpopulation in comparison to their corresponding Vδ2 T cell subpopulation.

### PD-1, TIM-3, and CD39 Were Co-expressed With TIGIT on Vδ1 T Cells in AML and MM

Since we found that PD-1, TIGIT, TIM-3, and CD39 are more frequently expressed by the Vδ1 subpopulation in AML and MM, the γδ T cells were further assessed with regard to a multiple co-expression of these co-regulatory molecules.

**Figure 5:**
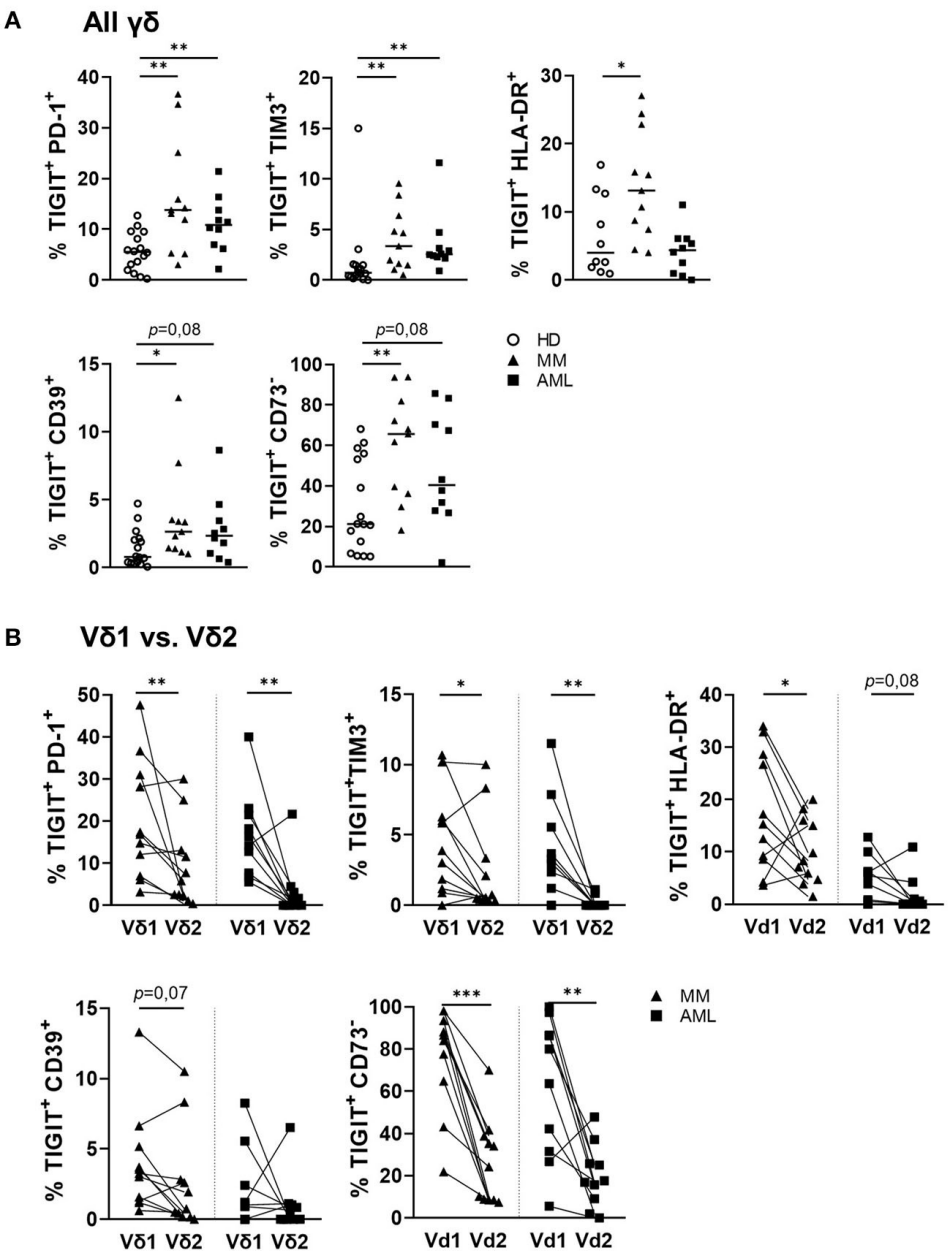
PD-1 and TIM-3 are co-expressed with TIGIT on γδ T cells in AML and MM. The co-expression of PD-1, TIGIT, TIM-3, CD39, CD73, and HLA-DR was compared between γδ T cells from peripheral blood (PB) of healthy donors (HD, white circles, *n* = 16), and bone marrow (BM) aspirates from patients with multiple myeloma (MM, black triangles, *n* = 11), and newly diagnosed AML (AML, black rectangles, *n* = 10). **(A)** Summary data are illustrating the co-expression of PD-1, TIM-3, CD39, CD73, and the HLA-DR receptor with TIGIT on γδ T cells. *P*-values were obtained by the Mann-Whitney test. *P < 0.05, **P < 0.01, ***P < 0.001. **(B)** Summary data show the co-expression of PD-1, TIM-3, CD39, CD73, and HLA-DR with TIGIT on paired Vδ1 and Vδ2 γδ T cells from patients with MM (black triangles) and AML (black rectangles). *P*-values were obtained by the Wilcoxon matched-pairs signed-rank test. *P < 0.05, **P < 0.01, ***P < 0.001.

The frequency of γδ T cells co-expressing PD-1, TIM-3, and CD39 together with TIGIT was significantly higher in samples from AML and MM in comparison to HDs (AML vs. HD: *p* = 0.006, *p* = 0.001, *p* = 0.08 and MM vs. HD: *p* = 0.004, *p* = 0.004, *p* = 0.017; Figure 5A). This difference of co-expression in AML and MM was caused by the significant co-expression of PD-1 and TIM-3 on the TIGIT^+^ Vδ1 γδ T cells in comparison to their corresponding Vδ2 cells (AML: *p* = 0.01, *p* = 0.008 and MM: *p* = 0.01, *p* = 0.042; Figure 5B). In contrast to the increased co-expression of TIGIT and CD39 in all γδ T cells in AML and MM, the clustering of CD39 on Vδ1 in comparison to the corresponding Vδ2 γδ T cell subset was only nearly significant in MM, and not significant in AML. In addition, the majority of TIGIT^+^ Vδ1 γδ T cells did not express the ectoenzyme CD73 (Figures 5A,B). Again, this multiple co-expression of co-inhibitory molecules was in particular observed on the Vδ1 CD27^−^CD45RA^++^ subset (Supplementary Figure 8).

In summary, the BM-infiltrating γδ T cells co-expressed PD-1, TIM-3, and CD39 with TIGIT in both hematological malignancies. Moreover, Vδ1 cells in AML and MM co-expressed PD-1 and TIM-3 with TIGIT more frequently than their corresponding Vδ2 T cells. For CD39, this was only the case in MM but not in AML CD39 was also significantly co-expressed with TIGIT on the Vδ1 T cells.

## Discussion

The present study provides a phenotypic analysis of the BM-resident γδ population in patients with AML and MM. Here, we demonstrate that Vδ1 T cells represent the predominant γδ T-cell population in the BM from these patients whereas Vδ2 T cells were dominant in the PB of HDs. The BM-infiltrating Vδ1 T cells in AML and MM showed an increased TEMRA cell compartment with an aberrant subpopulation of CD27^−^CD45RA^++^ cells in comparison to HDs. Expression analyses of corresponding immune effector cells derived from the BM revealed an increased expression of TIGIT, PD-1, TIM-3, and CD39 on γδ TCR cells in comparison to CD4^+^ cells, which was similar to that on CD8^+^ effector cells in both hematologic malignancies. In contrast to the Vδ2 T cells, the increased frequency of PD-1, TIGIT, TIM-3, and CD39 positive cells was mainly observed on Vδ1 T cells. This upregulated expression of co-regulatory receptors in AML and MM was related to the TEMRA γδ subpopulation and within this population, highest expression was found on the CD27^−^CD45RA^++^ cells. Furthermore, γδ T cells in AML and MM exhibited a further feature of exhaustion, manifested by an increased co-expression of multiple co-inhibitory molecules including PD-1, TIM-3, and CD39 together with TIGIT, which was caused by the increased co-expression on the Vδ1 T-cell population. In contrast, CD73 expression was downregulated by the TIGIT^+^ γδ T cells in AML and MM. Regarding the co-expression of multiple co-inhibitory receptors, we observed similar frequencies of double positive (TIGIT^+^PD-1^+^ / TIGIT^+^TIM-3^+^ or TIGIT^+^CD39^+^) cells in both cancer entities. In contrast, the single expression of TIGIT and HLA-DR was higher in MM, whereas TIM-3 was more frequently expressed by γδ T cells derived from patients with AML. These differences between both entities might be explained by variations in the immune response upon chronic stimulation.

γδ T cells are endowed with two independent recognition systems including the γδ TCR and NK cell receptors to identify tumor cells and initiate anti-cancer effector mechanisms, including cytokine production and cytotoxicity. Therefore, γδ T cells represent ideal effectors with a better safety profile and a more favorable anti-tumor efficacy in comparison to conventional αβ T cells because of their HLA-independent recognition of phosphoantigens that are characteristic for dysregulated metabolism in tumors (43, 44). Furthermore, the HLA- independent recognition system results in reduced graft vs. host disease and diminished target effects (44–47). Provided with these special features, γδ T cells represent attractive effector cells for adoptive T cell strategies. A recently designed γδ T cell construct with a GD2-targeted CAR showed potent responses against Disialoganglioside (GD2)^+^ neuroblastoma (48, 49). Moreover, first clinical studies have been conducted to investigate the safety and efficacy of adoptive transfer of autologous or allogeneic γδ T cells in cancer patients including renal cell carcinoma, lung cancer, hepatocellular carcinoma, breast cancer, prostate cancer, and multiple myeloma (50). These studies confirmed that γδ T cell application is safe, with low levels of adverse events and that the clinical responses are ranging from partial to complete remissions (50). In AML, promising data have been recently presented by Ganesan et al. demonstrating increased *in vitro* and *in vivo* γδ T cell-mediated cytotoxicity by a bispecific engager molecule against T cell receptor gamma variable 9 (TRGV9) and CD123 (51). However, there is still potential for improvement including the engagement of co-regulatory checkpoint molecules.

In agreement with other studies, we found that in BM from AML and MM patients, the infiltration of tissue-resident Vδ1 T cells was increased, whereas the frequency of the circulating Vδ2 T cells was lower compared to that in the PB from HDs (3, 52). This inversion of the Vδ1/Vδ2 ratio in different tissues has been recently observed in solid cancer including melanoma, colorectal cancer, and non-small cell lung cancer as well (52). Although a clear correlation with γδ T cell infiltration and prognosis of cancer patients is still missing, some studies demonstrated that a reduction of Vδ2 T cells correlates with an advanced stage. In line with this, enhanced levels of Vδ2 T cells correlated with an early stage of melanoma, the absence of metastasis and a longer 5-year disease-free survival rate of colorectal cancer patients (52).

To exclude that the differences observed between HD-derived PB and patient-derived BM could be compartment-associated, we additionally compared paired BM and PB aspirates from nine AML patients on a random basis. We observed no difference in the frequency of the total γδ T cells, nor in the distribution of Vδ1 and Vδ2 T cells. Moreover, no variations of checkpoint expression on γδ T cells and subpopulations respectively, were observed in these nine AML patients. Similar with our findings, Rossol et al. compared Vδ1 T cells derived from the BM and PB from patients with HIV and observed no significant differences (53). Dean et al.'s studies confirmed these observations, revealing no significant differences of γδ T cells when they compared aspirates from the BM and PB derived from patients with an active hematopoietic malignancy or in remission (54). Our study has the limitation of a relatively small sample size of patients with newly diagnosed AML and MM. Therefore, the observations made in this study should be interpreted with caution until validated in a larger cohort.

Our analyses of the differentiation status revealed a shift toward increased TEMRA differentiation. Migration of TEMRAs into inflammatory sites to perform effector functions has also been observed for chronic infections such as CMV, but also for solid cancer including neuroblastoma, colorectal cancer, and melanoma (5). For AML patients, Gertner-Dardenne et al. first described an increased memory profile in γδ T cells derived from the PB and BM, but in contrast to our study, they observed a differentiation into CD27^−^CD45RA^−^ defined EMs (6). To our knowledge, our study is the first description of the differentiation status in the BM for MM. We also found a unique γδ T cell subpopulation in the BM niche from patients with AML and MM that is characterized by the CD27^−^CD45RA^++^ phenotype. This aberrant subpopulation discovered by Odaira et al. is reported as a predominant subpopulation in different types of cancer and is characterized as “exhausted” subgroup defined by diminished proliferation capacity (41).

We and others have previously described an increased expression of TIGIT, PD-1, TIM-3, and CD39 on αβ T cells in AML and MM (31, 32, 55–57). Expression of these markers on αβ T cells was related to features of exhaustion manifested by transcriptional reprogramming, reduced effector cytokine production, decreased proliferation, and impaired lysis of tumor cells (32, 55, 58). Our comparative analyses of the co-regulatory marker expression on the corresponding CD4^+^, CD8^+^, and γδ T cells discovered that γδ T cells in the BM from patients with AML and MM exhibit a similar expression profile of TIGIT, PD-1, TIM-3, and CD39 to that on CD8^+^ T cells. In MM, γδ T cells showed even higher frequencies of TIGIT^+^ cells than the CD8^+^ population. Moreover, this study illustrated the increased co-expression of PD-1 and TIM-3 together with TIGIT on γδ T cells in AML and MM for the first time, hypothesizing that these cells are functionally “exhausted.”

Although the knowledge of checkpoint expression on γδ T cells is still sparse, our observations of an increased frequency of TIGIT^+^ γδ T cells confirm the data of increased levels of TIGIT^+^ γδ T cells in *de novo* AML patients published by Jin et al. In their study, upregulation of TIGIT on γδ T cells was associated with a lower overall survival rate for non-M3 AML (38). To our knowledge, there is no publication of the TIGIT expression on γδ T cells in MM.

PD-1 expression on human healthy γδ T cells has been reported upon antigen-stimulation (59). Additionally, the PD-L1/PD-1 signaling in γδ T cells prevented αβ T cell activation via checkpoint receptor ligation in pancreatic adenocarcinoma (60). In AML, pembrolizumab treatment in combination with zoledronate and interleukin-2 (IL-2) leads to increased IFN-γ production in γδ T cells *in vitro* (61). Also, in MM PD-1 expression increased in γδ T cells after zoledronate stimulation in contrast to HD-derived γδ T cells, suggesting that MM-derived γδ T cells are intrinsically programmed to increase their threshold of refractoriness via PD-1 upregulation. Interestingly, this upregulation was observed especially in the central memory subset of the Vδ2 T cells, which in normal conditions is the subset with the highest proliferative capacity (20).

Similar to TIGIT and PD-1, receptors, TIM-3 expression and function on conventional αβ T cells has been profoundly studied, but characterization on γδ T cells is very limited. Schofield et al. reported an upregulation of TIM-3 on γδ T cells in childhood malaria. This was also mainly observed in the TEMRA population and was regulated by IL-18 and IL-12 (62). In colorectal cancer, the fraction of TIM-3^+^ γδ T cells has been reported to be increased, which correlated with tumor-lymph-node-metastasis (TNM) staging. Moreover, TIM-3 signaling significantly inhibited the killing efficiency of Vδ2 T cells against colon cancer cells and reduced the secretion of perforin and granzyme B (63).

CD39^+^ expression has been reported on tissue-resident γδ T cells (64). Moreover, CD39^+^ γδ T cells suppressed immune responses via the adenosine pathway by recruitment of myeloid-derived suppressor cells in colorectal cancer (39). These CD39^+^ γδ T cells also expressed FOXP3^+^, a marker of regulatory αβ T cells but have more potent immunosuppressive activity than CD4^+^ or CD8^+^ regulatory T cells (Tregs) (39). Moreover, Casetti et al. reported that FOXP3^+^ γδ T cells have the potential to inhibit the proliferation of anti-CD3/anti-CD28 stimulated PBMCs *in vitro* (65).

Despite Vδ1 and Vδ2 T cells both having cytotoxic capability, it has been shown that these two subsets express distinct chemokine receptors and cell adhesion molecules (1, 12, 15, 66, 67). Our data revealed that increased expression of TIGIT, PD-1, TIM-3, and CD39 was mainly confined to the Vδ1 T cell subpopulation in the BM from AML and MM patients. Recent studies found that the less studied Vδ1 T cells outperform Vδ2 T cells in most *in vitro* and first *in vivo* cancer models in terms of cytotoxicity and cell persistence after allogeneic cell transfer (68). Moreover, because of their different chemokine-receptor profile, Vδ1 T cells exhibit an increased ability of tissue penetration in comparison to Vδ2 T cells (69). Those results underline the functional relevance of the observed upregulation of multiple co-regulatory receptors on these cytotoxic effector Vδ1 T cells in AML and MM in this study.

### Conclusion

This study identified γδ T cells, in particular Vδ1 T cells, in AML and MM as expressors of multiple inhibitory receptors including TIGIT, PD-1, TIM-3, and the potential tissue residency marker CD39. (Co-)expression of inhibitory receptors may reflect their exhaustion status and represent targetable structures on cytotoxic effector cells. Inhibition of TIGIT, PD-1, TIM-3, and CD39 especially on Vδ1 T cells alone or in combinatorial strategies should be further analyzed with regard to re-invigorating and boosting their cytotoxicity.

## Data Availability Statement

The original contributions presented in the study are included in the article/Supplementary Material, further inquiries can be directed to the corresponding author.

## Ethics Statement

The studies involving human participants were reviewed and approved by Declaration of Helsinki and approval by the local ethics board of the Ärztekammer Hamburg (PV3469 and PV5119). The patients/participants provided their written informed consent to participate in this study.

## Author Contributions

FB designed the research study, analyzed the data, and wrote the manuscript. PW performed the experiments, analyzed the data, and reviewed the manuscript. KW, LL, and BF provided the myeloma aspirates and reviewed the manuscript. CB and JSzW reviewed the manuscript. JW and WF conceived the concept, oversaw the interpretation and presentation of the data, and reviewed the manuscript. All authors read and approved the final manuscript.

## Funding

FB was supported by the Mildred Scheel Nachwuchszentrum Hamburg and the Hamburg Translational Research in Cancer program at the University Medical Center Hamburg-Eppendorf, Hamburg, Germany. JSzW was funded by the SFB1328 and by the DFG SFB841. The project was funded by the Roggenbuck Stiftung.

## Conflict of Interest

FB: Travel grant Daiichi Sankyo, Servier, Novartis; advisory board by Jazz. GmbH, Daiichi Sankyo. WF: Membership on an entity's board of directors or advisory Amgen, ARIAD/Incucyte, Pfizer, Novartis, Jazz Pharmaceuticals, Morphosys, Abbvie, Celgene; patents and royalities: Amgen; other support for meeting attendance Amgen, Gilead, Jazz Pharmaceuticals, Servier, Daiichi Sankyo; research funding Amgen, Pfizer. Travel grant, advisory board and research funding by Amgen Inc., travel grant and advisory board by TEVA GmbH, the advisory board: Ariad/Incucyte Inc., travel grant by Gilead Inc and Jazz. GmbH, research funding by Pfizer Inc. KW: Honoraria: AbbVie, Amgen, Adaptive Biotech, Celgene/BMS, GSK, Janssen, Karyopharm, Novartis, Roche, Takeda, Sanofi; Research funding: Amgen, Celgene, Janssen, Sanofi. LL: Non-financial support from GSK and from Abbvie. CB: Travel grant: Astra Zeneca, Bayer Healthcare, Berlin Chemie, Bristol Myers Squipp, Jansen Cilag, Merck Serono, Merck Sharp Dohme, Novartis, Roche Pharma, Sanofi Aventis; Advisory board: Astra Zeneca, Bayer Healthcare, Berlin Chemie, Bristol Myers Squipp, Jansen Cilag, Merck Serono, Merck Sharp Dohme, Novartis, Roche Pharma, Sanofi Aventis; Invited speaker: AOK Germany, med update, Merck Serono; Honoraria: AOK Germany, Astra Zeneca, Bayer Healthcare, Berlin Chemie, GSO Research Organisation, Jansen Cilag, med update, Merck Serono, Merck Sharp Dohme, Novartis, Roche Pharma, Sanofi Aventis. The remaining authors declare that the research was conducted in the absence of any commercial or financial relationships that could be construed as a potential conflict of interest. The handling editor declared a past co-authorship with the authors JW and WF.

## Publisher's Note

All claims expressed in this article are solely those of the authors and do not necessarily represent those of their affiliated organizations, or those of the publisher, the editors and the reviewers. Any product that may be evaluated in this article, or claim that may be made by its manufacturer, is not guaranteed or endorsed by the publisher.

## Abbreviations

ADO: adenosine
aHSCT: allogeneic hematopoietic stem cell transplantation
AML: Acute myeloid leukemia
ATP: adenosine triphosphate
BiTEs: bispecific T cell engagers
BM: bone marrow
CAR-T cells: chimeric antigen receptor T cells
CD39: ectonucleoside triphosphate diphosphohydrolase-1
CD73: ecto-5′-nucleotidase
CM: central memory cells
CMV: cytomegalovirus
EM: effector memory cells
DC: dendritic cells
FMO: Fluorescence minus one
HD: healthy donor
IFN-γ: Interferon-γ
IL-2: interleukin-2
IL-10: interleukin 10
IL-12: interleukin 12
MM: multiple myeloma
MCF: multiparametric flow cytometry
MFI: mean fluorescence intensity
MGUS: monoclonal gammopathy of undetermined significance
NA: naïve cells
NKG2D: natural killer group 2D
non-APL AML: non-acute promyelocytic leukemia
PB: peripheral blood
PVR: poliovirus receptor
PVRL2: poliovirus receptor-related 2
TEMRA: terminal differentiated effector memory cells
TIGIT: T cell immunoreceptor with Ig and ITIM domains
TIL: tumor-infiltrating lymphocytes
TIM-3: hepatitis A virus cellular receptor 2
TME: tumor microenvironment
TNF: tumor necrosis factor
tSNE: t-distributed stochastic neighbor embedding.
