# Supplementary material for: Bone Marrow-Resident Vδ1 T Cells Co-express TIGIT With PD-1, TIM-3 or CD39 in AML and Myeloma
Source: Front Med (Lausanne). 2021 Nov 8;8:763773. doi: 10.3389/fmed.2021.763773 (PMC8606547; doi:10.3389/fmed.2021.763773)
Supplement: Supplementary file 1 [file Presentation_1.pptx]

## Slide 1
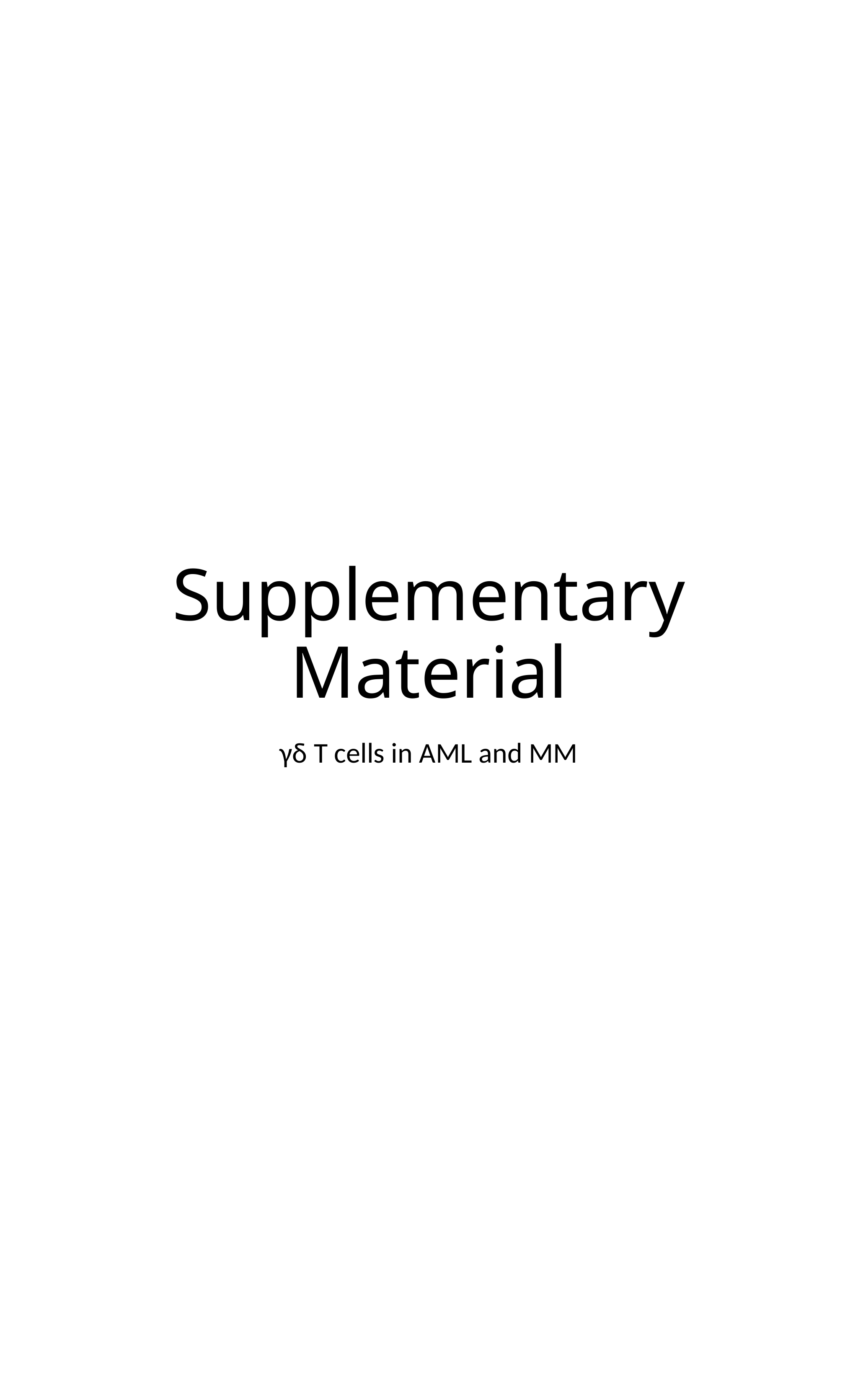

# Supplementary Material
γδ T cells in AML and MM

## Slide 2
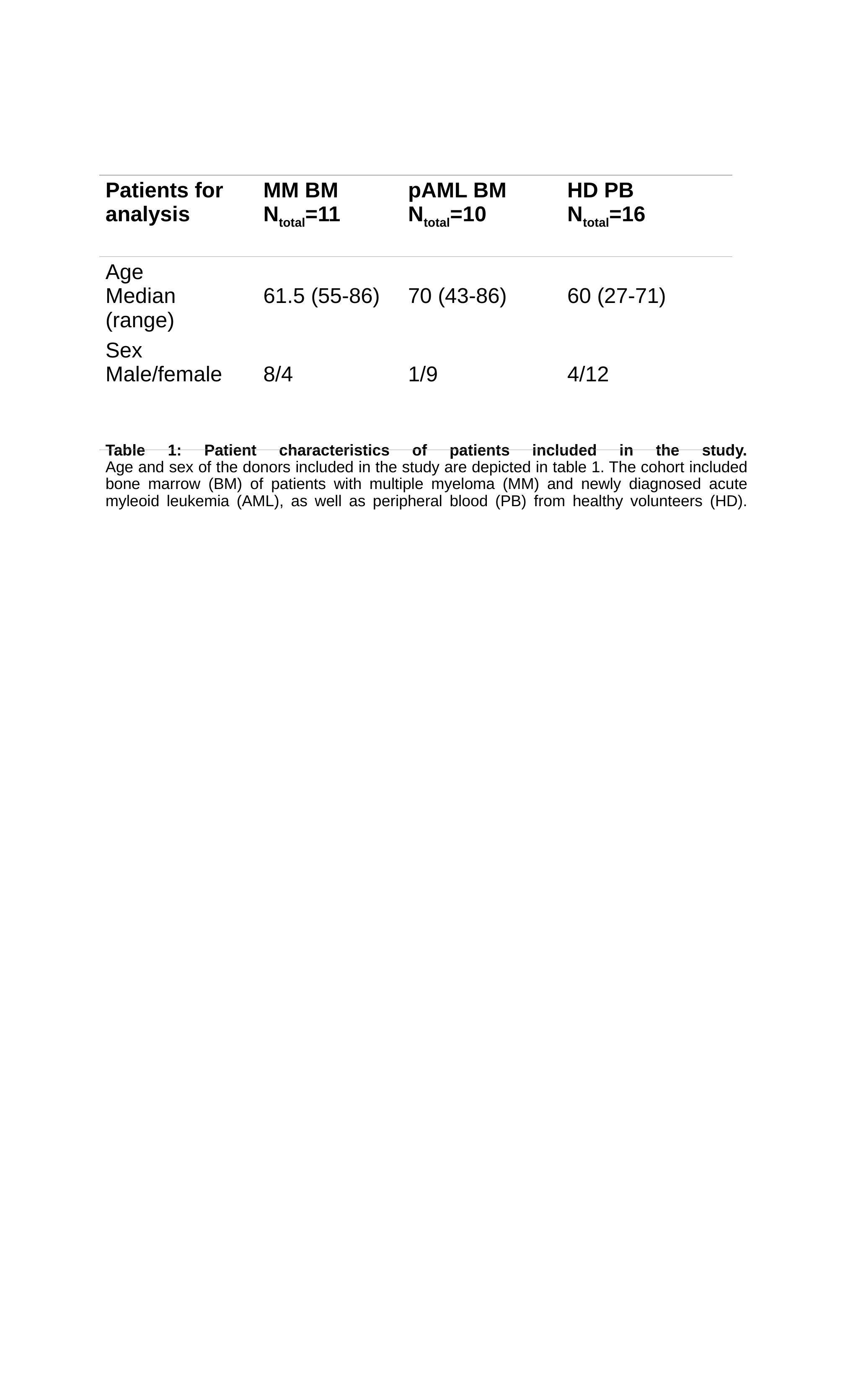

| Patients for analysis | MM BM Ntotal=11 | pAML BM Ntotal=10 | HD PB Ntotal=16 | |
| --- | --- | --- | --- | --- |
| Age Median (range) | 61.5 (55-86) | 70 (43-86) | 60 (27-71) | |
| Sex Male/female | 8/4 | 1/9 | 4/12 | |
| | | | | |
| | | | | |
# Table 1: Patient characteristics of patients included in the study. Age and sex of the donors included in the study are depicted in table 1. The cohort included bone marrow (BM) of patients with multiple myeloma (MM) and newly diagnosed acute myleoid leukemia (AML), as well as peripheral blood (PB) from healthy volunteers (HD).

## Slide 3
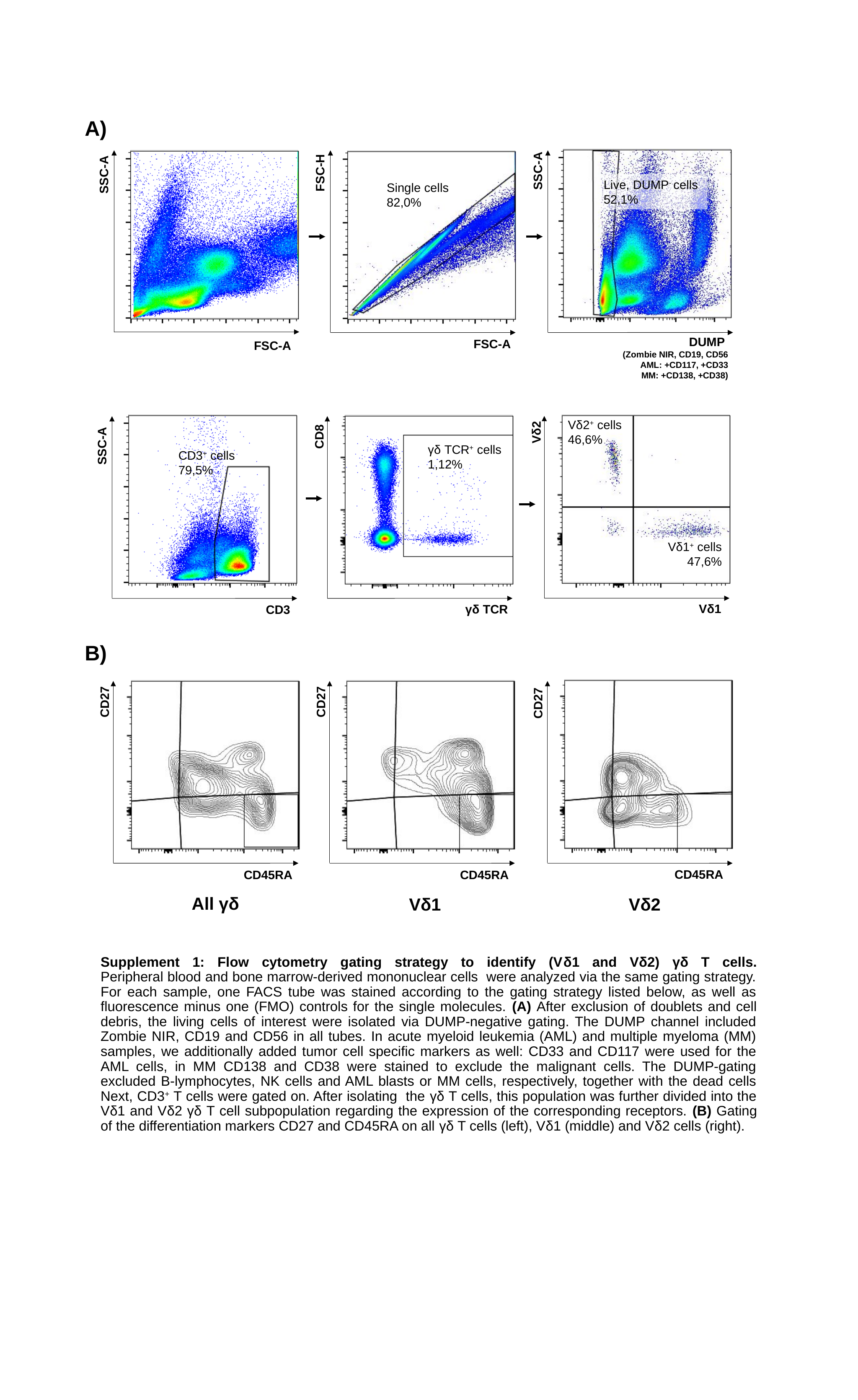

A)
SSC-A
FSC-H
SSC-A
Live, DUMP- cells 52,1%
Single cells
82,0%
DUMP
(Zombie NIR, CD19, CD56
AML: +CD117, +CD33
MM: +CD138, +CD38)
FSC-A
FSC-A
SSC-A
CD3+ cells
79,5%
CD3
Vδ2
Vδ2+ cells
46,6%
CD8
γδ TCR+ cells
1,12%
Vδ1+ cells
47,6%
Vδ1
γδ TCR
B)
CD27
CD45RA
CD27
CD27
CD45RA
CD45RA
All γδ
Vδ1
Vδ2
# Supplement 1: Flow cytometry gating strategy to identify (Vδ1 and Vδ2) γδ T cells. Peripheral blood and bone marrow-derived mononuclear cells were analyzed via the same gating strategy. For each sample, one FACS tube was stained according to the gating strategy listed below, as well as fluorescence minus one (FMO) controls for the single molecules. (A) After exclusion of doublets and cell debris, the living cells of interest were isolated via DUMP-negative gating. The DUMP channel included Zombie NIR, CD19 and CD56 in all tubes. In acute myeloid leukemia (AML) and multiple myeloma (MM) samples, we additionally added tumor cell specific markers as well: CD33 and CD117 were used for the AML cells, in MM CD138 and CD38 were stained to exclude the malignant cells. The DUMP-gating excluded B-lymphocytes, NK cells and AML blasts or MM cells, respectively, together with the dead cells Next, CD3+ T cells were gated on. After isolating the γδ T cells, this population was further divided into the Vδ1 and Vδ2 γδ T cell subpopulation regarding the expression of the corresponding receptors. (B) Gating of the differentiation markers CD27 and CD45RA on all γδ T cells (left), Vδ1 (middle) and Vδ2 cells (right).

## Slide 4
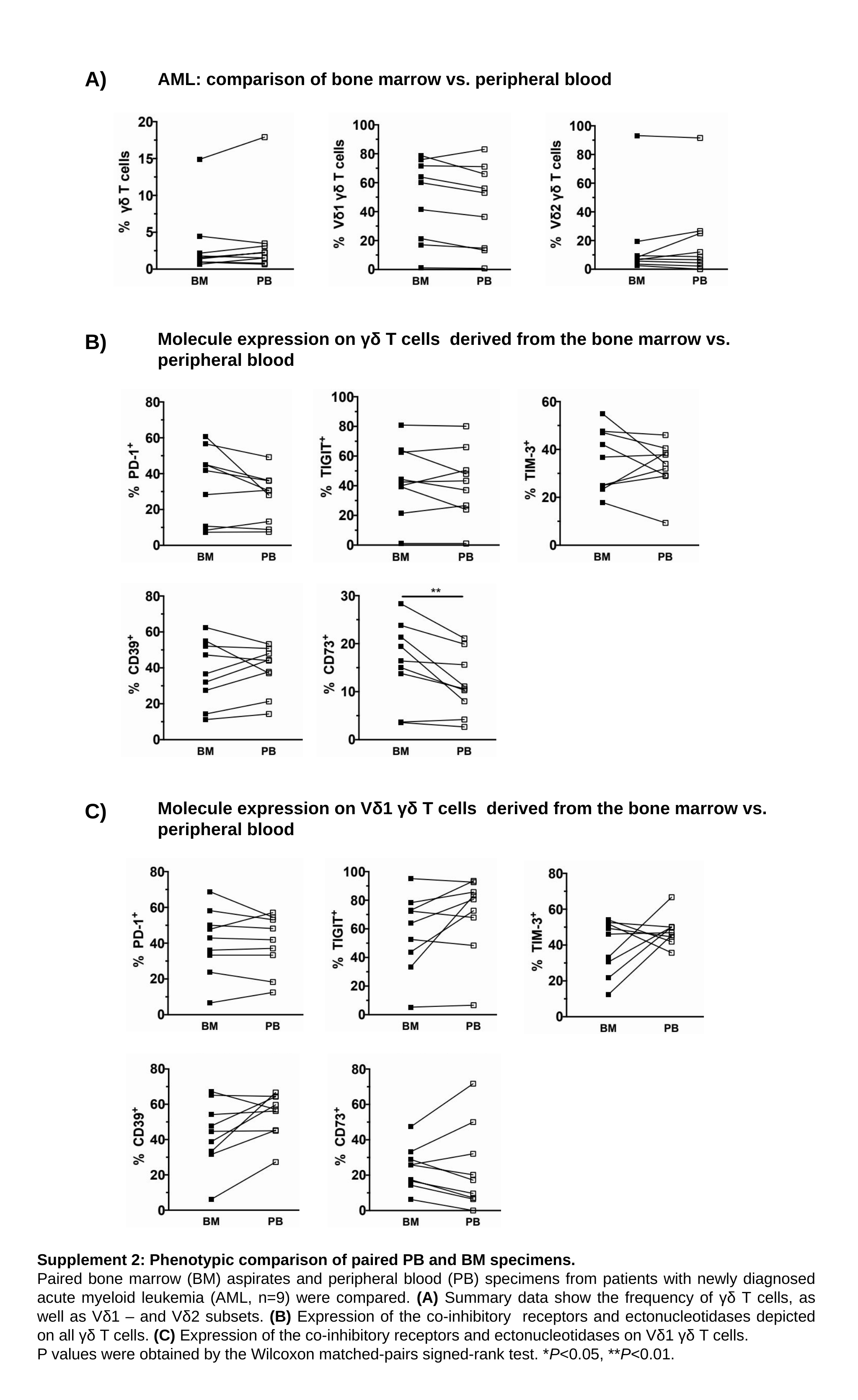

A)
AML: comparison of bone marrow vs. peripheral blood
Molecule expression on γδ T cells  derived from the bone marrow vs. peripheral blood
B)
Molecule expression on Vδ1 γδ T cells  derived from the bone marrow vs. peripheral blood
C)
Supplement 2: Phenotypic comparison of paired PB and BM specimens.
Paired bone marrow (BM) aspirates and peripheral blood (PB) specimens from patients with newly diagnosed acute myeloid leukemia (AML, n=9) were compared. (A) Summary data show the frequency of γδ T cells, as well as Vδ1 – and Vδ2 subsets. (B) Expression of the co-inhibitory receptors and ectonucleotidases depicted on all γδ T cells. (C) Expression of the co-inhibitory receptors and ectonucleotidases on Vδ1 γδ T cells.
P values were obtained by the Wilcoxon matched-pairs signed-rank test. *P<0.05, **P<0.01.

## Slide 5
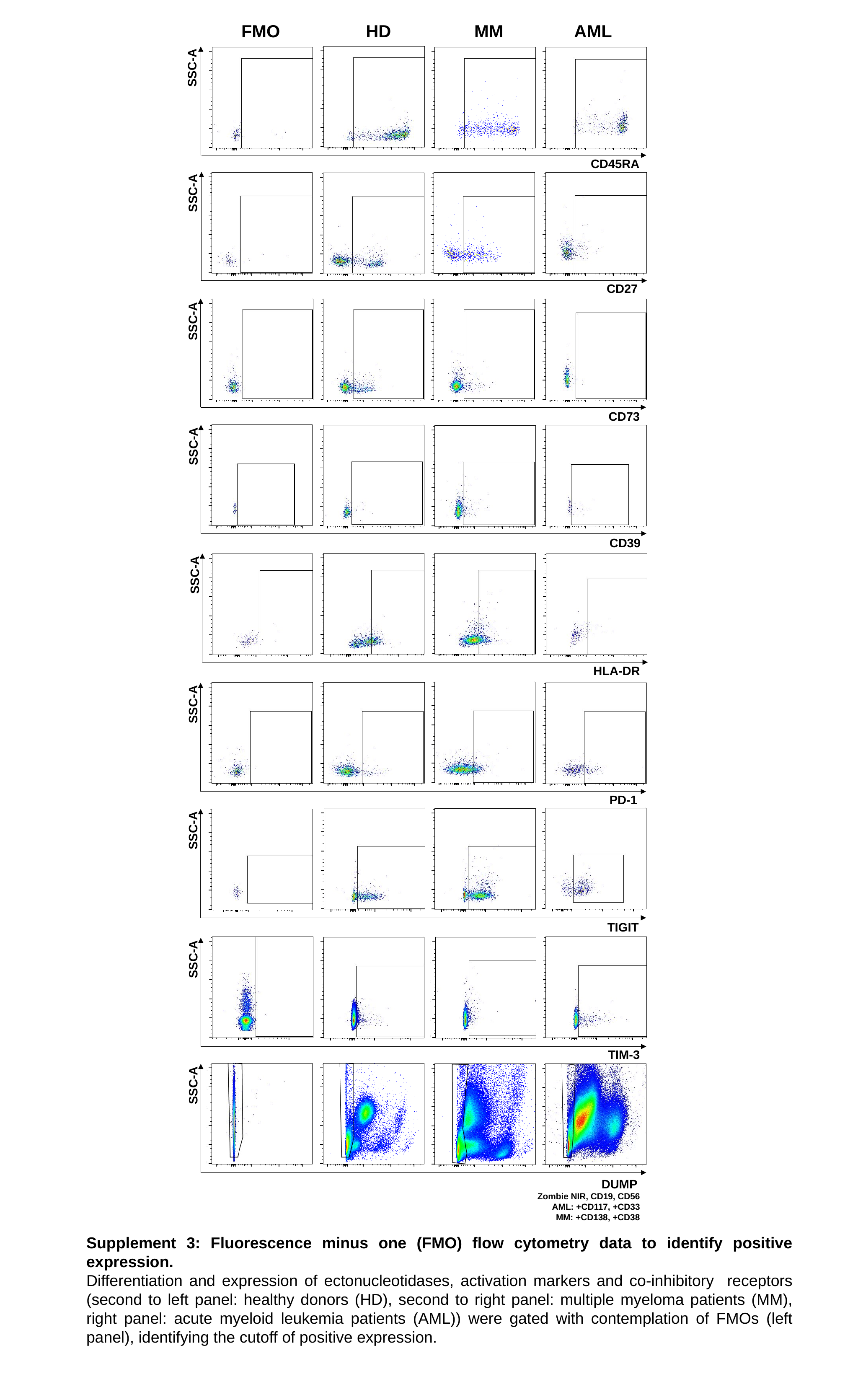

# FMO
HD
MM
AML
SSC-A
CD45RA
SSC-A
CD27
SSC-A
CD73
SSC-A
CD39
SSC-A
HLA-DR
SSC-A
PD-1
SSC-A
TIGIT
SSC-A
TIM-3
SSC-A
DUMP
Zombie NIR, CD19, CD56
AML: +CD117, +CD33
MM: +CD138, +CD38
Supplement 3: Fluorescence minus one (FMO) flow cytometry data to identify positive expression.
Differentiation and expression of ectonucleotidases, activation markers and co-inhibitory receptors (second to left panel: healthy donors (HD), second to right panel: multiple myeloma patients (MM), right panel: acute myeloid leukemia patients (AML)) were gated with contemplation of FMOs (left panel), identifying the cutoff of positive expression.

## Slide 6
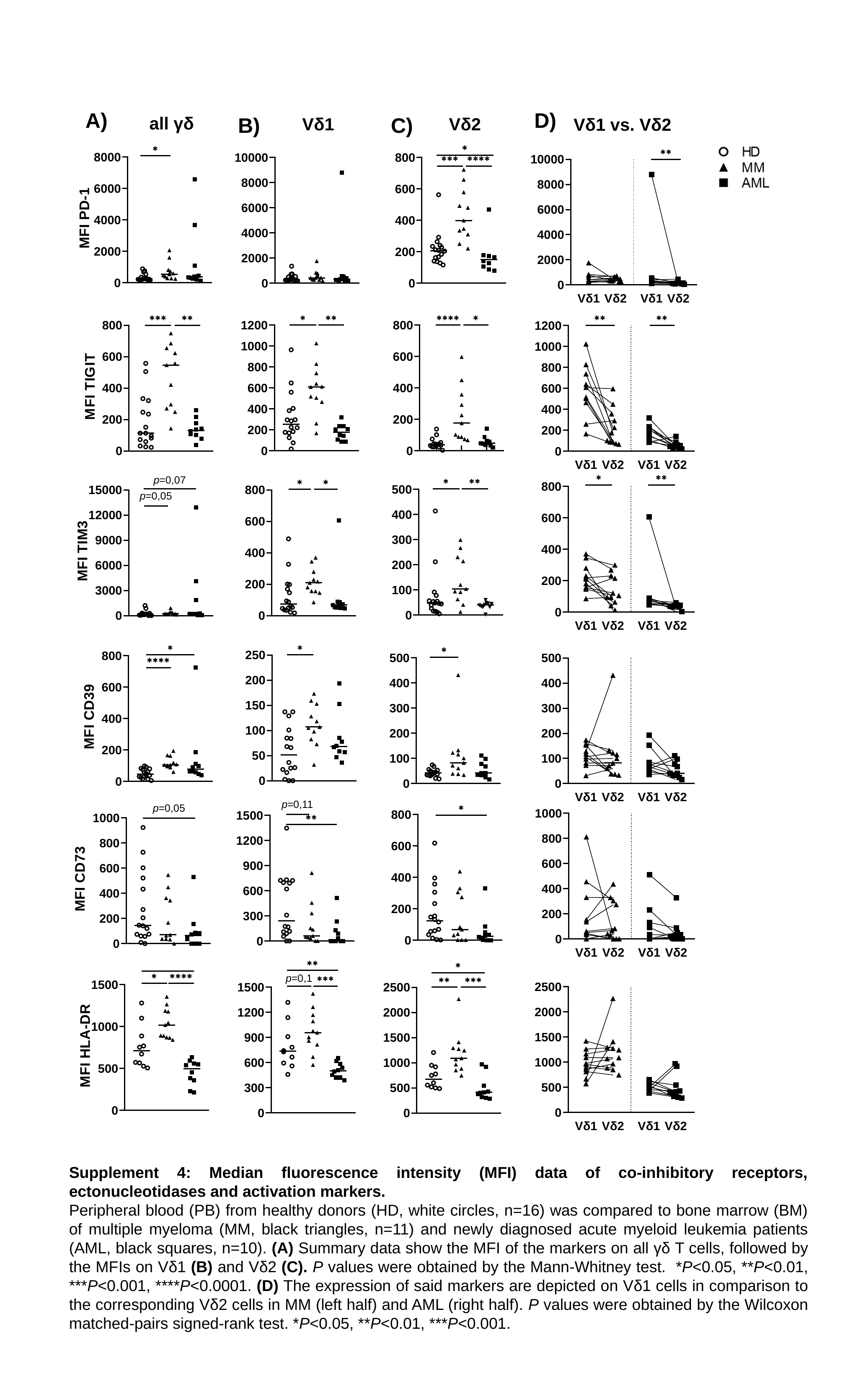

D)
A)
B)
C)
all γδ
Vδ1
Vδ2
Vδ1 vs. Vδ2
Supplement 4: Median fluorescence intensity (MFI) data of co-inhibitory receptors, ectonucleotidases and activation markers.
Peripheral blood (PB) from healthy donors (HD, white circles, n=16) was compared to bone marrow (BM) of multiple myeloma (MM, black triangles, n=11) and newly diagnosed acute myeloid leukemia patients (AML, black squares, n=10). (A) Summary data show the MFI of the markers on all γδ T cells, followed by the MFIs on Vδ1 (B) and Vδ2 (C). P values were obtained by the Mann-Whitney test. *P<0.05, **P<0.01, ***P<0.001, ****P<0.0001. (D) The expression of said markers are depicted on Vδ1 cells in comparison to the corresponding Vδ2 cells in MM (left half) and AML (right half). P values were obtained by the Wilcoxon matched-pairs signed-rank test. *P<0.05, **P<0.01, ***P<0.001.

## Slide 7
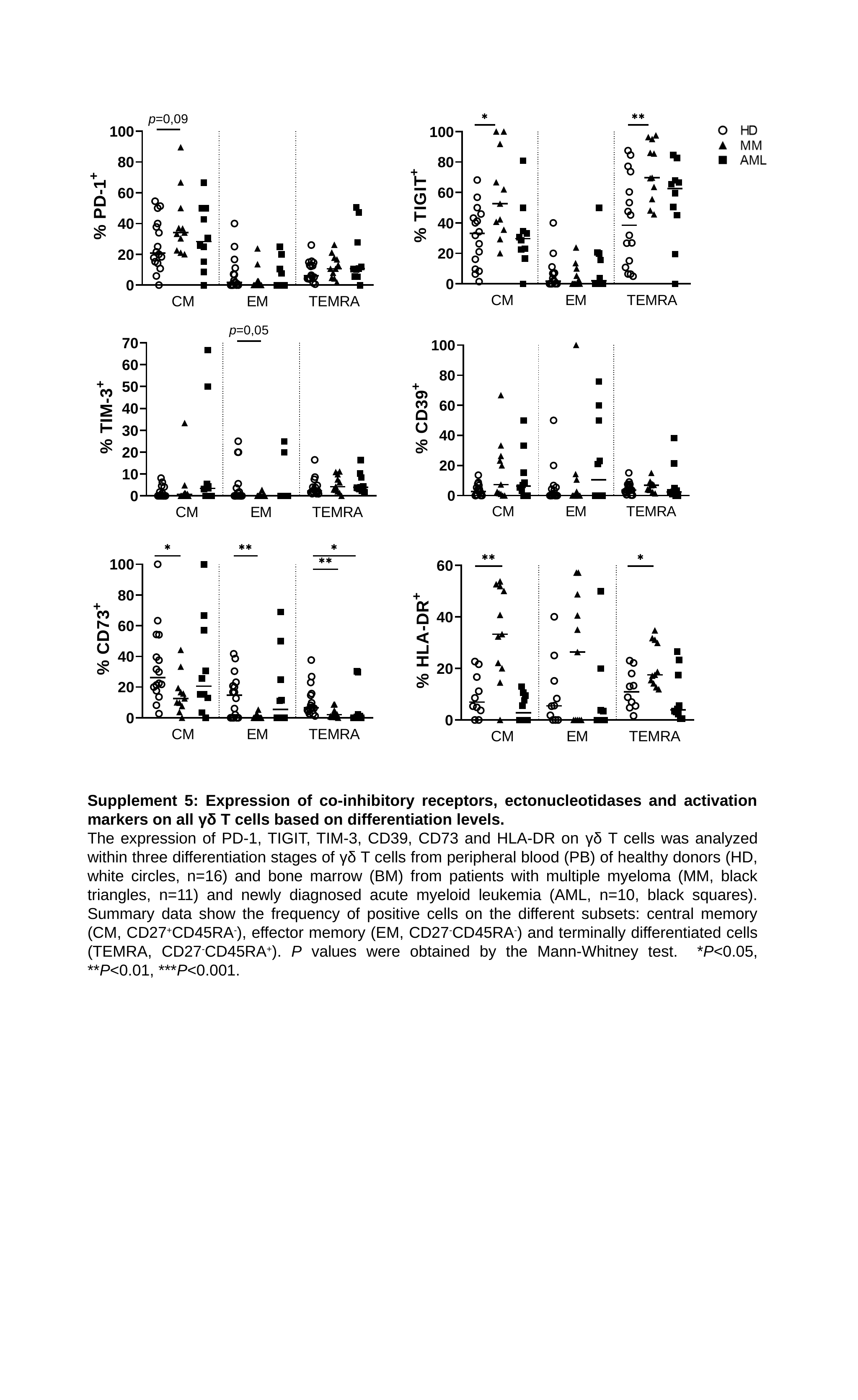

Supplement 5: Expression of co-inhibitory receptors, ectonucleotidases and activation markers on all γδ T cells based on differentiation levels.
The expression of PD-1, TIGIT, TIM-3, CD39, CD73 and HLA-DR on γδ T cells was analyzed within three differentiation stages of γδ T cells from peripheral blood (PB) of healthy donors (HD, white circles, n=16) and bone marrow (BM) from patients with multiple myeloma (MM, black triangles, n=11) and newly diagnosed acute myeloid leukemia (AML, n=10, black squares). Summary data show the frequency of positive cells on the different subsets: central memory (CM, CD27+CD45RA-), effector memory (EM, CD27-CD45RA-) and terminally differentiated cells (TEMRA, CD27-CD45RA+). P values were obtained by the Mann-Whitney test. *P<0.05, **P<0.01, ***P<0.001.

## Slide 8
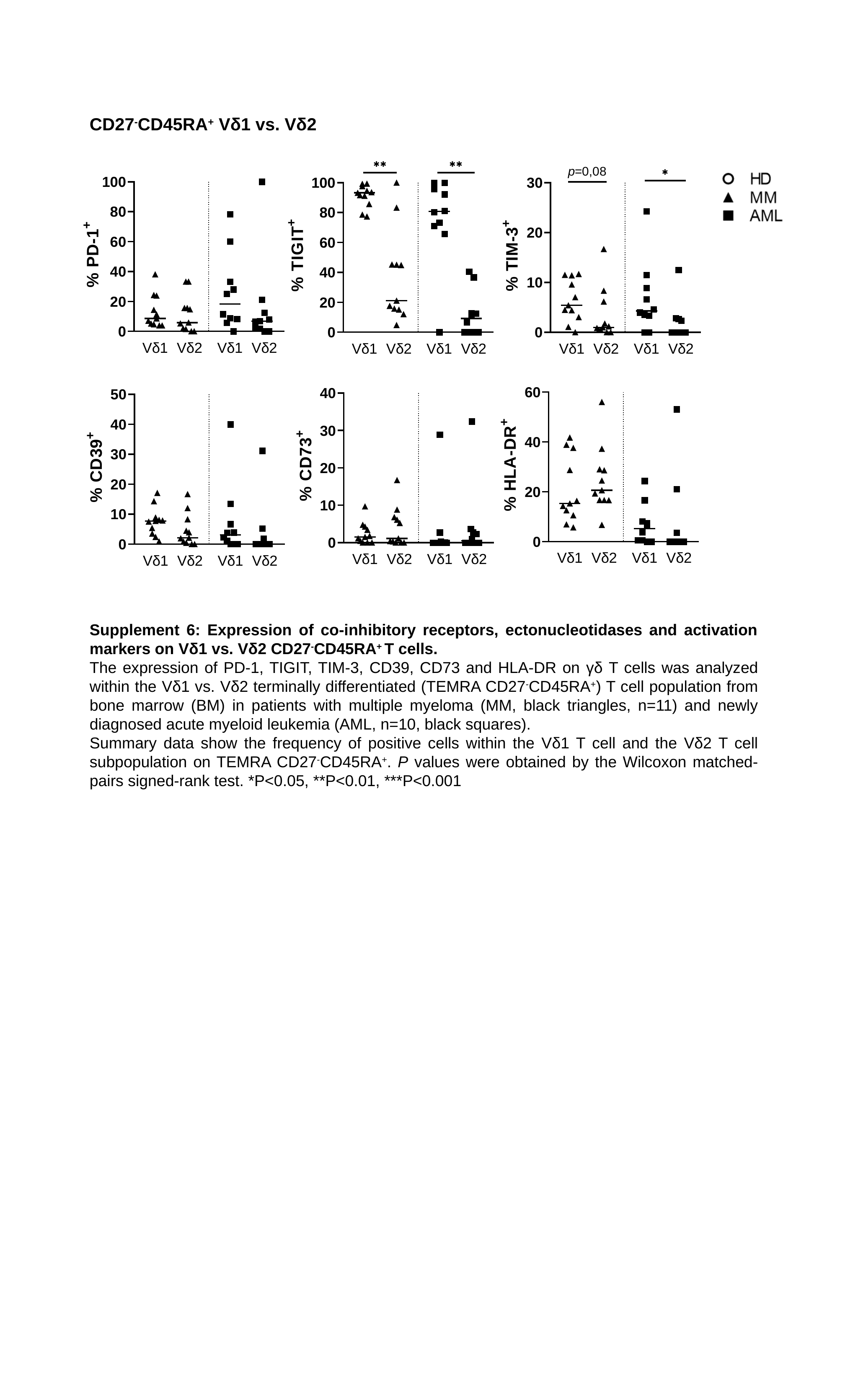

CD27-CD45RA+ Vδ1 vs. Vδ2
Supplement 6: Expression of co-inhibitory receptors, ectonucleotidases and activation markers on Vδ1 vs. Vδ2 CD27-CD45RA+ T cells.
The expression of PD-1, TIGIT, TIM-3, CD39, CD73 and HLA-DR on γδ T cells was analyzed within the Vδ1 vs. Vδ2 terminally differentiated (TEMRA CD27-CD45RA+) T cell population from bone marrow (BM) in patients with multiple myeloma (MM, black triangles, n=11) and newly diagnosed acute myeloid leukemia (AML, n=10, black squares).
Summary data show the frequency of positive cells within the Vδ1 T cell and the Vδ2 T cell subpopulation on TEMRA CD27-CD45RA+. P values were obtained by the Wilcoxon matched-pairs signed-rank test. *P<0.05, **P<0.01, ***P<0.001

## Slide 9
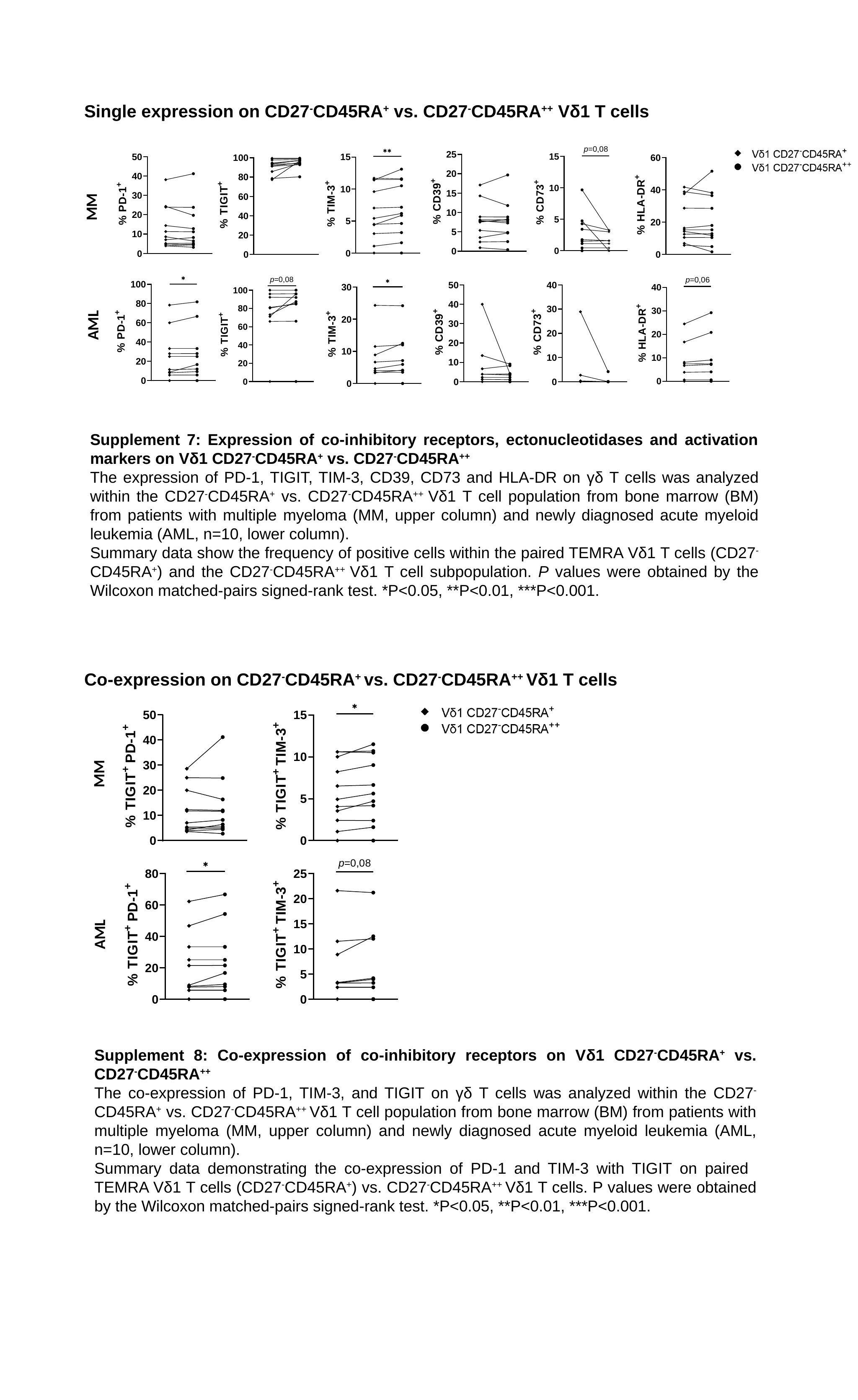

Single expression on CD27-CD45RA+ vs. CD27-CD45RA++ Vδ1 T cells
MM
AML
Supplement 7: Expression of co-inhibitory receptors, ectonucleotidases and activation markers on Vδ1 CD27-CD45RA+ vs. CD27-CD45RA++
The expression of PD-1, TIGIT, TIM-3, CD39, CD73 and HLA-DR on γδ T cells was analyzed within the CD27-CD45RA+ vs. CD27-CD45RA++ Vδ1 T cell population from bone marrow (BM) from patients with multiple myeloma (MM, upper column) and newly diagnosed acute myeloid leukemia (AML, n=10, lower column).
Summary data show the frequency of positive cells within the paired TEMRA Vδ1 T cells (CD27-CD45RA+) and the CD27-CD45RA++ Vδ1 T cell subpopulation. P values were obtained by the Wilcoxon matched-pairs signed-rank test. *P<0.05, **P<0.01, ***P<0.001.
Co-expression on CD27-CD45RA+ vs. CD27-CD45RA++ Vδ1 T cells
MM
AML
Supplement 8: Co-expression of co-inhibitory receptors on Vδ1 CD27-CD45RA+ vs. CD27-CD45RA++
The co-expression of PD-1, TIM-3, and TIGIT on γδ T cells was analyzed within the CD27-CD45RA+ vs. CD27-CD45RA++ Vδ1 T cell population from bone marrow (BM) from patients with multiple myeloma (MM, upper column) and newly diagnosed acute myeloid leukemia (AML, n=10, lower column).
Summary data demonstrating the co-expression of PD-1 and TIM-3 with TIGIT on paired TEMRA Vδ1 T cells (CD27-CD45RA+) vs. CD27-CD45RA++ Vδ1 T cells. P values were obtained by the Wilcoxon matched-pairs signed-rank test. *P<0.05, **P<0.01, ***P<0.001.
